# Glycomic Analysis of Human Respiratory Tract Tissues and Correlation with Influenza Virus Infection

**DOI:** 10.1371/journal.ppat.1003223

**Published:** 2013-03-14

**Authors:** Trevenan Walther, Rositsa Karamanska, Renee W. Y. Chan, Michael C. W. Chan, Nan Jia, Gillian Air, Clark Hopton, Maria P. Wong, Anne Dell, J. S. Malik Peiris, Stuart M. Haslam, John M. Nicholls

**Affiliations:** 1 Department of Pathology, The University of Hong Kong, Queen Mary Hospital, Pokfulam, Hong Kong, China; 2 Division of Molecular Biosciences, Faculty of Natural Sciences, Biochemistry Building, Imperial College London, South Kensington Campus, London, United Kingdom; 3 Centre of Influenza Research, School of Public Health, Li Ka Shing Faculty of Medicine, The University of Hong Kong, Pokfulam, Hong Kong, China; 4 Department of Biochemistry & Molecular Biology, University of Oklahoma Health Sciences Center, Oklahoma City, Oklahoma, United States of America; 5 HKU-Pasteur Research Centre, Hong Kong, China; Johns Hopkins University - Bloomberg School of Public Health, United States of America

## Abstract

The first step in influenza infection of the human respiratory tract is binding of the virus to sialic (Sia) acid terminated receptors. The binding of different strains of virus for the receptor is determined by the α linkage of the sialic acid to galactose and the adjacent glycan structure. In this study the N- and O-glycan composition of the human lung, bronchus and nasopharynx was characterized by mass spectrometry. Analysis showed that there was a wide spectrum of both Sia α2-3 and α2-6 glycans in the lung and bronchus. This glycan structural data was then utilized in combination with binding data from 4 of the published glycan arrays to assess whether these current glycan arrays were able to predict replication of human, avian and swine viruses in human *ex vivo* respiratory tract tissues. The most comprehensive array from the Consortium for Functional Glycomics contained the greatest diversity of sialylated glycans, but was not predictive of productive replication in the bronchus and lung. Our findings indicate that more comprehensive but focused arrays need to be developed to investigate influenza virus binding in an assessment of newly emerging influenza viruses.

## Author Summary

This study was performed to determine what possible glycan receptors for influenza were present in the human respiratory tract. We compared the glycans present on existing published glycan arrays with the actual glycans identified in the human respiratory tract by mass spectrometric analysis to determine how representative these arrays would be for potential binding. The most comprehensive array to date only contained approximately half the range of the actual glycans present. Over the past 5 years we have performed *ex-vivo* infection of 113 bronchial and 185 lung samples with seasonal, avian and swine influenza viruses, and have demonstrated that the lung is able to be infected by all types of influenza viruses but that the bronchus can also be infected by a limited range of avian, swine and seasonal viruses. The key findings are that there is wide spectrum of glycans present in the respiratory tract which can be used by influenza viruses for infection, and the currently available arrays are not predictive of successful infection. Our findings will be of use for researchers in developing more comprehensive and focused arrays for the screening of emerging influenza viruses and bacteria in order to determine their potential threat to humans.

## Introduction

Influenza virus infection in humans presents an economic and social health burden to society. Yearly infections are normally due to H1N1, H3N2 or influenza B strains while pandemics occur at 30–40 year intervals due to antigenic shift or the emergence of new strains, such as the introduction of H1N1 of swine origin into the human population which occurred in 2009.

The natural reservoir of influenza virus are waterfowl species, in which all subtypes of influenza virus can be found and cause asymptomatic infection. Nevertheless, the influenza virus also infects mammalian species, for example horses, swine and humans, in which several subtypes are able to establish their lineage in the population. The universal ligand for all influenza viruses is sialic acid (Sia) linked to galactose, but since the 1980s the 2 main types of influenza viruses – avian and human have been distinguished by the configuration of the bond between the Sia and galactose, with viruses infecting humans having a preference to bind the Sia-Gal in an α2-6 configuration and those infection binds with an α2-3 configuration [1], [2].

Previous studies have been conducted to examine the distribution of receptors in the human respiratory tract, so as to predict the virus binding tropism. Using lectin histochemistry, it was demonstrated in the 1990's that the human upper respiratory tract including the trachea appeared to contain mainly α2-6 receptors (reviewed in [1]). It was thus considered that avian viruses with α2-3 binding such as H5N1 or H7N7 (which can cause a high mortality) would not normally infect the upper respiratory tract of humans unless there was a shift from α2-3 to α2-6. Swine have been proposed as a mixing vessel of human and avian influenza virus as lectin binding studies indicated that their respiratory tract contained both α2-3 and α2-6 linked receptors [2]. In 1997, the H5N1 avian virus emerged in Hong Kong where it infected humans directly without the need for passing though an intermediate host and with retention of its α2-3 binding specificity. Further lectin binding studies in the human respiratory tract demonstrated that the lower respiratory tract, especially the lung had mainly α2-3 linked receptors which was thought to explain this predilection for the lower respiratory tract [3]. Additional studies using different isoforms of the lectin *Maackia amurensis* indicated that the upper respiratory tract of humans also contains α2-3 linked receptors and they could also serve as a site for replication of H5N1 [4]. This finding inferred that the lectins used for binding are not able to determine the fine glycan structure of the respiratory tract. Thus, if there was a diversity of sialylated glycans present in the human respiratory tract, a single lectin binding assay may not be enough to identify all the potential receptors present along the respiratory tract. This pinpointed a strong need in the field to have a comprehensive study of the sialylated glycans in the respiratory tract.

In 2004, a printed glycan array was produced in which large numbers of glycans were included to allow a more comprehensive analysis of binding preference of different strains of influenza virus and to evaluate whether binding was simply a matter of α2-6 versus α2-3 [4], [5], [6]. To date there are four main glycan arrays being used to evaluate the relation between influenza infection and glycan binding preference and specificity. The most comprehensive one has been developed by the Consortium for Functional Glycomics, and contains over 600 glycans in version 5. A modified form of the CFG array is utilized by the Center for Disease Control and Prevention while Ten Feizi and colleagues in United Kingdom, and Wong and colleagues in Taiwan have also developed their own glycan arrays [7], [8]. Though these array platforms provide valuable information on influenza virus binding, the problem has been in determining the clinical significance of their results, as the spectrum of glycans present in the respiratory tract was not known. Therefore, studies on the glycan binding profiles of influenza viruses using these glycan arrays have not been able to distinguish glycans present in the respiratory tract (and therefore of physiological relevance to influenza biology) and those that are not expressed in the respiratory tract (thus irrelevant for influenza virology).

Because of this lack of detailed knowledge of the actual glycans present in the respiratory tract, the first aim of this study was to identify the range of sialylated glycans, and thus possible receptor profiles present in different regions of the respiratory tract of humans. With this information, our second aim was to determine to what extent the 4 available arrays were representative of the actual glycans present. As we had tissues from the bronchus and lung available for glycan analysis as well as ex vivo infection [9], [10], [11], the third aim was to see if there was a difference in the replication of a diverse range of influenza viruses, and if there was a difference, to determine if this difference was due to glycan composition or other factors. In particular we wished to investigate if a difference in regional infection was a matter of the α2-6 versus α2-3 linkage or whether other components of the glycan affected efficient replication. The outline of the study design is highlighted in Figure S1.

## Results

### Glycomic characterization of human respiratory tract tissues shows a wide range of N and O-linked glycans

**Figure 1:**
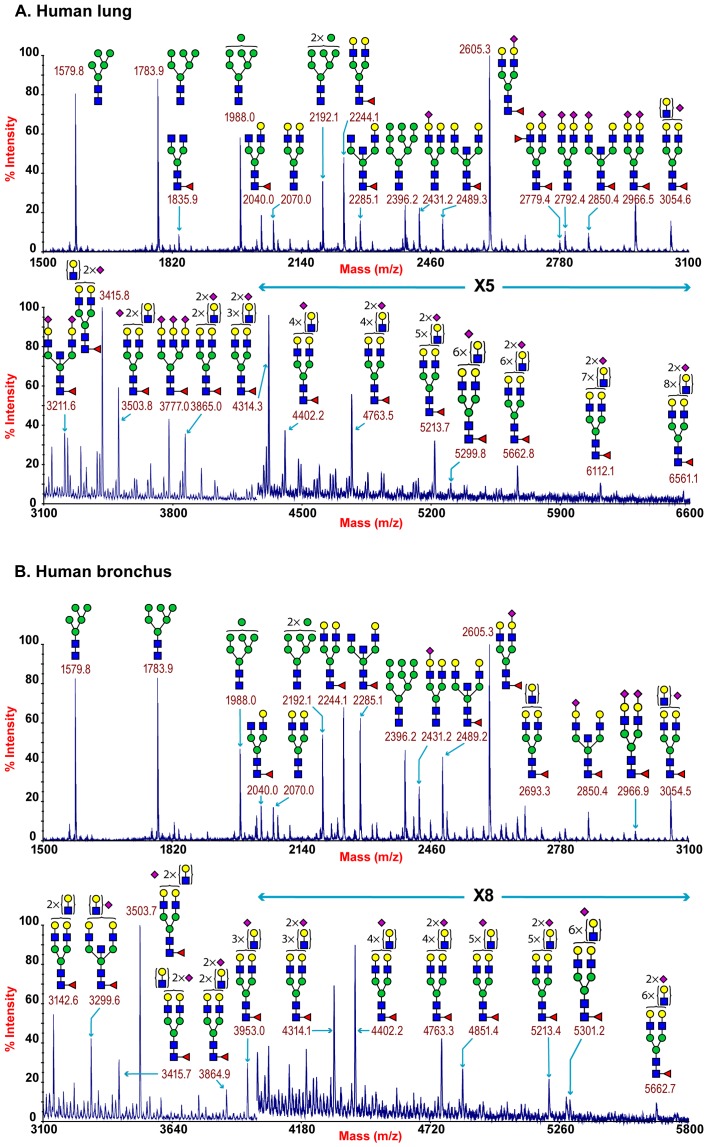
N-glycan profile of lung and bronchus. MALDI-TOF mass spectra of permethylated N-glycans of human lung and bronchus. N-Glycomic profiles of human lung (A) and human bronchus (B) were obtained from the 50% MeCN fraction from a C18 Sep-Pak column (“Materials and Methods”). Annotated structures are according to the Consortium for Functional Glycomics guidelines. All molecular ions are [M+Na]+. Putative structures are based on composition, tandem MS, and the biosynthetic knowledge. Due to the presence of heterogeneous multiantennary structures with extended LacNAc repeats, the annotations are simplified throughout by using biantennary structures with the extensions listed in parentheses. Structures that show sugars outside a bracket have not been unequivocally defined (see Table S1).

MALDI-TOF profiling of the permethylated N-glycans from the human lung afforded a spectrum rich in [M+Na]^+^ molecular ion signals up to m/z 6600 (Figure 1A). A series of complex glycans with compositions consistent with core fucosylated bi-, tri- and tetra-antennary structures bearing multiple LacNAc extensions were observed (NeuAc_0-4_Hex_5-13_HexNAc_4-12_Fuc_0-4_). Minor species with compositions consistent with the presence of bisecting GlcNAc were also present (for example m/z 3211 NeuAc2Hex5HexNAc5Fuc). The major non-reducing end-capping groups were the Galß1-4GlcNAc sequence and NeuAc-Gal-GlcNAc. Minor but significant levels of fucosylation were also observed making Lewis X (Galβ4[Fucα3]GlcNAc) structures (m/z 2779). In addition a full set of high mannose glycans were observed (m/z 1579-2396 Man_5-9_GlcNAc_2_) (Figure 1A, Table S1).

Additional levels of structural definition were afforded by MS/MS fragmentation of the major molecular ion species between m/z 2000–5000. All monofucosylated molecular ions gave a Y-ion at m/z 474 indication core fucosylation, while more heavily fucosylated structures also gave a B-ion at m/z 660 consistent with Lewis X termini. All molecular ions with a composition indicating the presence of siaylation produced B-ion at m/z 847 consistent with a NeuAc-Gal-GlcNAc capped antennae. This is illustrated from the MS/MS spectra of the molecular ion at m/z 4312 which has a composition of NeuAc_2_Hex_8_HexNAc_7_Fuc and produces fragment ions consistent with NeuAcLacNAc_1-3_ (Figure S2). This provides definitive evidence for the presence of heterogeneous mixtures of bi-, tri-, and tetra-antennary N-glycans with varying lengths of sialylted polyLacNAc extension in the human lung. Additional evidence for the presence of sialylted polyLacNAc extension in the human lung came from endo-ß-galactosidase digestion which produced a digested fragment at m/z 1085 NeuAcHexHexNAcHex (data not shown).

The total pool of lung derived N-linked glycans was also subject to GC-MS linkage analysis. The presence of 2-linked, 2,4- and 2,6-linked Man revealed the presence of bi-, tri-, and tetra-antennary complex N-glycans. 4,6-linked GlcNAc and 3,4,6-linked Man confirmed the presence of core fucosylated and bisected complex structures. Significant peaks for both 3- and 6-linked Gal indicated the presence of both α2–3- and α 2–6-sialylated glycans, though the 3-Gal will also be produced from LacNAc extensions (Table S2).

MALDI-TOF profiling of the permethylated N-glycans from the human adult bronchus again afforded a spectrum rich in [M+Na]^+^ molecular ion signals up to m/z 5900 (Figure 1B). Similar to the lung, a series of complex glycans with compositions consistent with core fucosylated bi-, tri- and tetra-antennary structures bearing multiple LacNAc extensions were observed (NeuAc_0-3_Hex_3-12_HexNAc_4-11_Fuc_0-3_). Minor species with compositions consistent with the presence of bisecting GlcNAc were also present (for example m/z 3299 NeuAc2Hex5HexNAc5Fuc). The major non-reducing end-capping groups were the Galß1-4GlcNAc and NeuAc-Gal-GlcNAc. Minor but significant levels of fucosylation were also observed consistent with Lewis X containing structures. In addition a full set of high mannose glycans were observed (m/z 1579-2396 Man_5-9_GlcNAc_2_) (Figure 1B, Table S3). Again MS/MS fragmentation of the major molecular ion species between m/z 2000–5000 was performed. All monofucosylated molecular ions gave a Y-ion at m/z 474 indication core fucosylation, whilst all molecular ions whose composition indicated the presence of sialylation produced B-ion at m/z 847 consistent with a NeuAc-Gal-GlcNAc capped antennae. As illustrated from the MS/MS spectra of the molecular ion at m/z 3054 which has a composition of NeuAc_1_Hex_6_HexNAc_5_Fuc, fragment ions consistent with NeuAcLacNAc_1-2_ was observed. This provides definitive evidence for the presence of heterogeneous mixtures of multi-antennary N-glycans with varying lengths of sialylated polyLacNAc extension in the human bronchus (Figure S3).

GC-MS linkage analysis of pooled bronchus N-glycans contained significant peaks for both 3- and 6-linked Gal indicating the presence of both α2–3- and α2–6-sialylated glycans, though the 3-Gal will also be produced from LacNAc extensions (Table S4).

**Figure 2:**
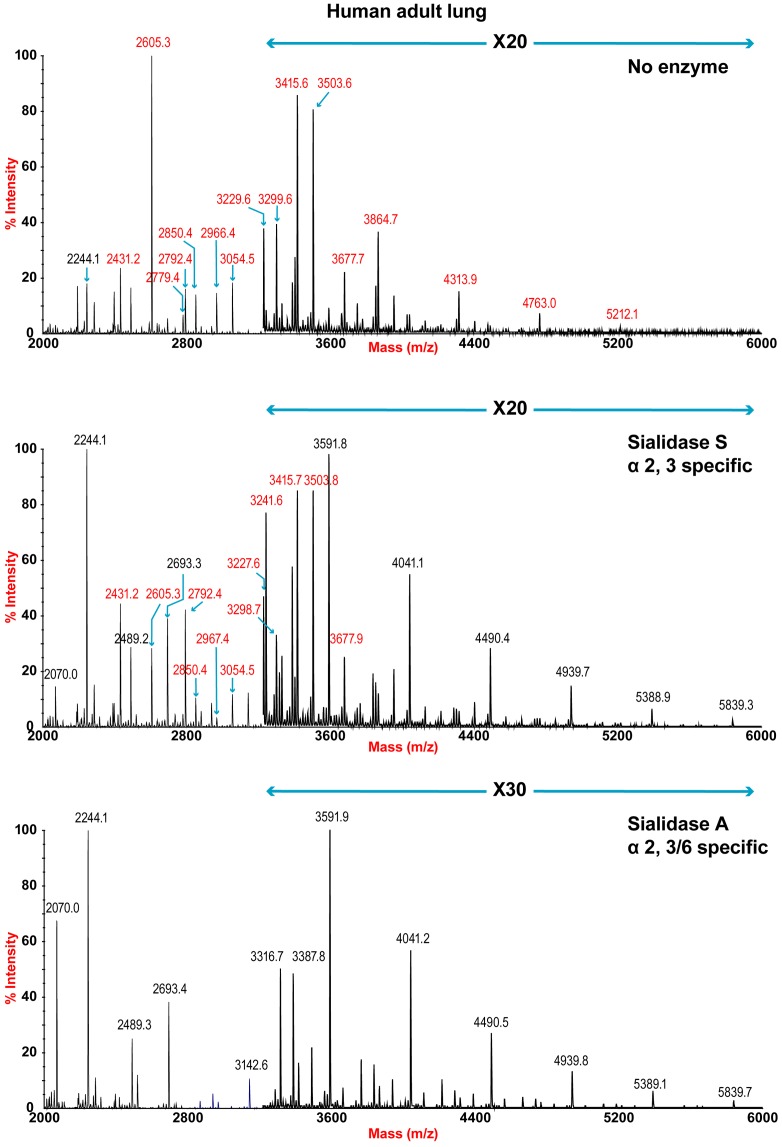
N-glycan profile of lung following sialidase treatment. Partial MALDI-TOF MS profiles of the permethylated N-linked glycans derived from human lung after digestion with sialidase S (specific release of α 2–3-linked Sia) or sialidase A (release of both α2–3- and α2–6-linked Sia). Data were obtained from the 50% acetonitrile fraction and all molecular ions are present in sodiated form ([M+Na]^+^). Sialylated species are annotated in red (see Table S1).

**Figure 3:**
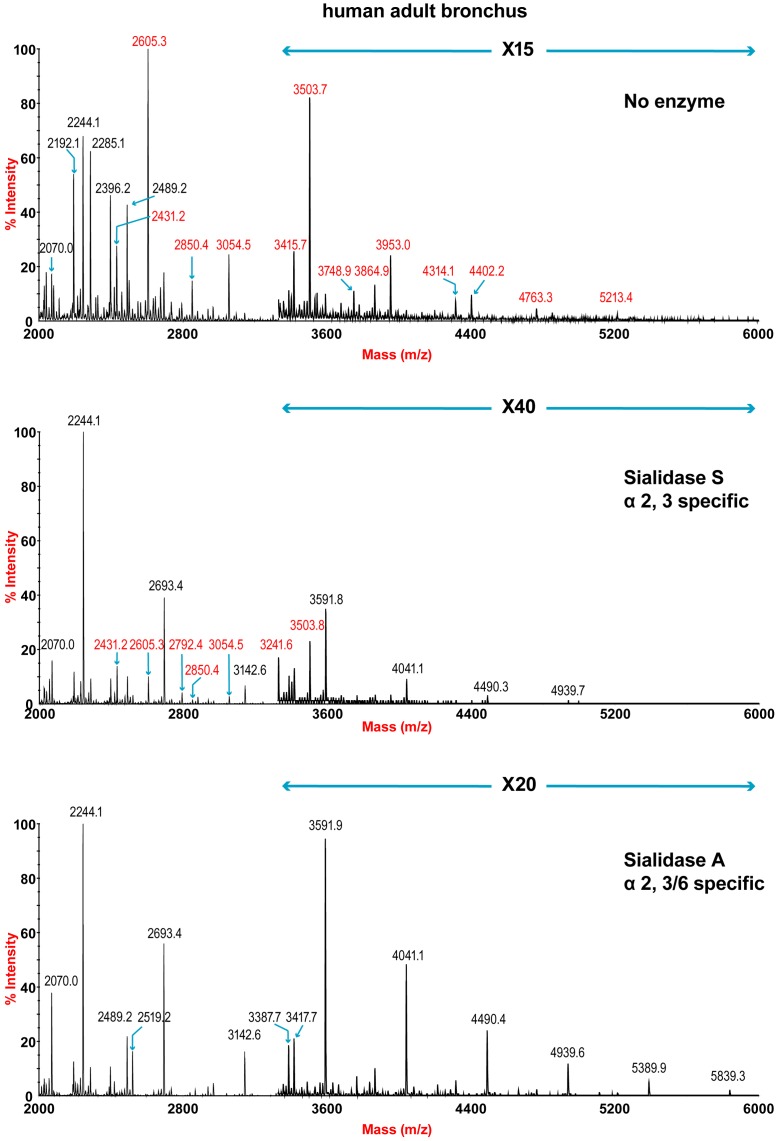
N-glycan profile of bronchus following sialidase treatment. Partial MALDI-TOF MS profiles of the permethylated N-linked glycans derived from human bronchus after digestion with sialidase S (specific release of α 2–3-linked Sia) or sialidase A (release of both α2–3- and α2–6-linked Sia). Data were obtained from the 50% acetonitrile fraction and all molecular ions are present in sodiated form ([M+Na]^+^). Sialylated species are annotated in red (see Table S1).

The sensitivity of the lung and bronchus to digestion with linkage-specific sialidases was examined by subsequent MALDI-TOF analysis. Sialidase S was used for the specific release of α 2–3-linked Sia and sialidase A for release of both α2–3- and α2–6-linked Sia. Digestion with linkage-specific sialidases indicated a comparable abundance of sialylated glycan structures with both α2-3 and α2-6 -linkage. Digestion of human lung N-glycans by sialidase A resulted in a complete loss of sialylated species (for example m/z 2605, 2966, 3415, and 4763). The spectra is dominated by compositions consistent with core fucosylated complex N-glycans with up to 10 LacNAc units (m/z 2244-5839), which again indicates that human lung contains heterogeneous mixtures of bi-, tri-, and tetraantennary N-glycans, with varying lengths of sialylted polyLacNAc extension. In contrast digestion of human lung N-glycans by sialidase S resulted in only partial desialylation, with sialylated N-glycans at m/z 2431, 2605, 2792, 3850 and 3054 still remaining prominent (Figure 2). Digestion of human bronchus N-glycans by sialidase A resulted in a complete loss of sialylated species (for example m/z 2605, 2966, 3415, and 4763). Again the spectra is dominated by compositions consistent with core fucosylated complex N-glycans with up to 10 LacNAc units (m/z 2244-5839). Digestion of human bronchus N-glycans by Sialidase S resulted in partial desialylation, with sialylated N-glycans at m/z 2431, 2605, 2792, 3850 and 3054 still remaining prominent (Figure 3). However in comparison to the human lung sample a higher proportion of sialylated N-glycans were digested. This indicated that both the human lung and bronchus contains mixtures of α2–3- and α2–6-linked Sia and that the bronchus contains more α2–3 Sia.

**Figure 4:**
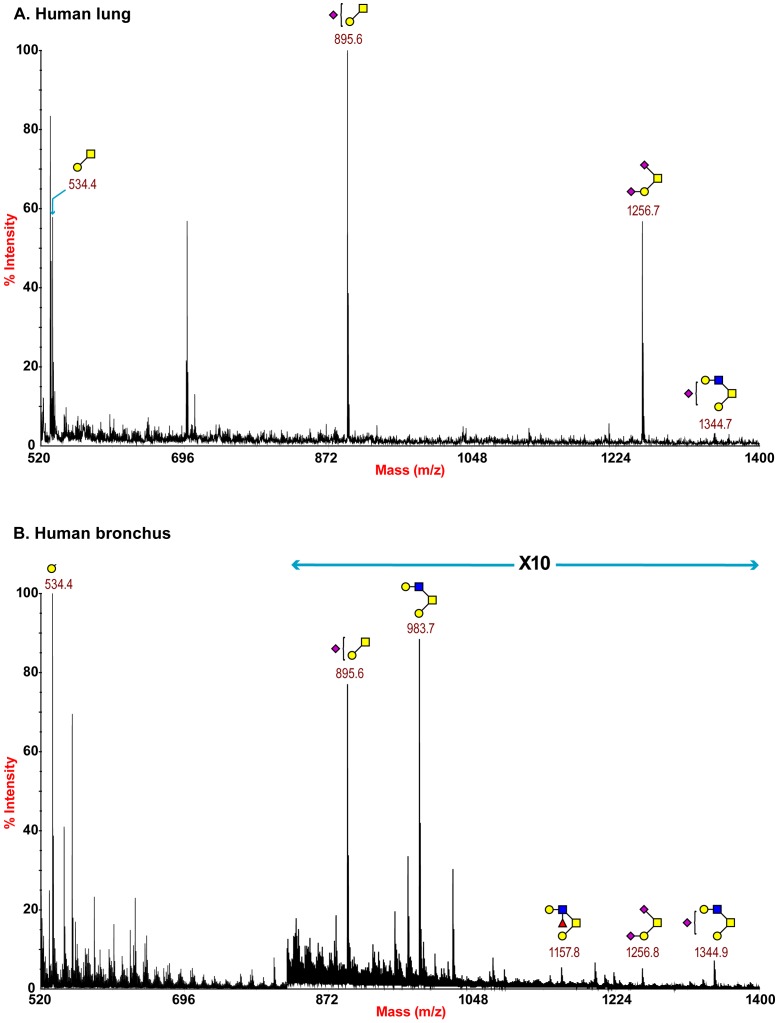
O-glycan profile of lung and bronchus. MALDI-TOF mass spectra of permethylated O-glycans of human lung and bronchus. O-Glycomic profiles of human lung (A) and human bronchus (B) were obtained from the 35% MeCN fraction from a C18 Sep-Pak column (“Experimental Procedures”). Annotated structures are according to the Consortium for Functional Glycomics guidelines. All molecular ions are [M+Na]+. Putative structures are based on composition, tandem MS, and the biosynthetic knowledge (see Table S2).

O-glycan structures of the lung and bronchus are shown in Figure 4. The O-glycan profile in human lung (Figure 4 and Table S5, S6) demonstrated the presence of both core 1 and core 2 structures, capped with NeuAc. For core 1, the main sialylated O-glycans were at m/z 895.6 and 1256.8 while the sialylated core 2 was at m/z 1344.7. O-glycan profile of the bronchus demonstrated the presence of both core 1 and core 2 structures, capped with NeuAc. MS/MS analysis of the mono-sialylated core 1 structure, m/z 895 NeuAcHexHexNAc, indicated that the sialic acid can be attached either to the Gal or GalNAc residue (data not shown).

The nasopharynx had a more limited range of glycans compared to the other human tissues with fewer extended LAcNAc profiles identified (Figure S4). However, it should be noted that a more limited amount of tissue for glycomic analysis was available which could inhibit the detection of more high molecular weight glycan species.

Similar to the adult lung, the paediatric lung expressed a complex pattern of N-glycans consisting off compositions consistent with core fucosylated bi-, tri- and tetra-antennary structures bearing multiple LacNAc extensions (NeuAc_0-2_Hex_5-9_HexNAc_4-8_Fuc_0-1_).and a full set of high mannose glycans (m/z 1579-2396 Man_5-9_GlcNAc_2_) (Figure S5, Table S7). Digestion with linkage-specific sialidase S (α2-3-linked) and sialidase A (both α2-3 and α2-6-linked) indicated the presence of Sia both α2-3 and α2-6-linked, with a greater abundance of α2-3 glycans than the adult lung. This was demonstrated by the complete loss of the sialylated glycans at m/z 4313.8, 3952.8, 3776.7, 3415.6, 2966.4, and 2850.4 and also the significant digestion of the sialylated glycans at m/z 3836.7, 3054.5, 2605.3 and 2431.2 (Figure S5).

The paediatric bronchus, similar to the adult bronchus, expressed a complex pattern of N-glycans consisting off compositions consistent with core fucosylated bi-, tri- and tetra-antennary structures bearing multiple LacNAc extensions (NeuAc_0-2_Hex_5-9_HexNAc_4-8_Fuc_0-1_) and a full set of high mannose glycans (m/z 1579-2396 Man_5-9_GlcNAc_2_) (Figure S6, Table S8). There was a reduction in the relative abundance of complex glycans, however, it should be noted that a more limited amount of tissue for glycomic analysis was available which could inhibit the detection of more high molecular weight glycan species. Digestion with linkage-specific sialidase S (α2-3-linked) and sialidase A (both α2-3 and α2-6-linked) indicated the presence of Sia both α2-3 and α2-6-linked, with a greater abundance of α2-3 glycan than the adult bronchus (Figure S6).

The O-glycan profiles of the paediatric tissues demonstrated the presence of both core 1 and core 2 structures, which are capped with NeuAc (Figure S7, Tables S9, S10). When compared to the O-glycan profile of adult tissues, additional core 2 structures (for example m/z 1705.7) were observed.

### Currently available array platforms identified glycans present in the human respiratory tract

**Figure 5:**
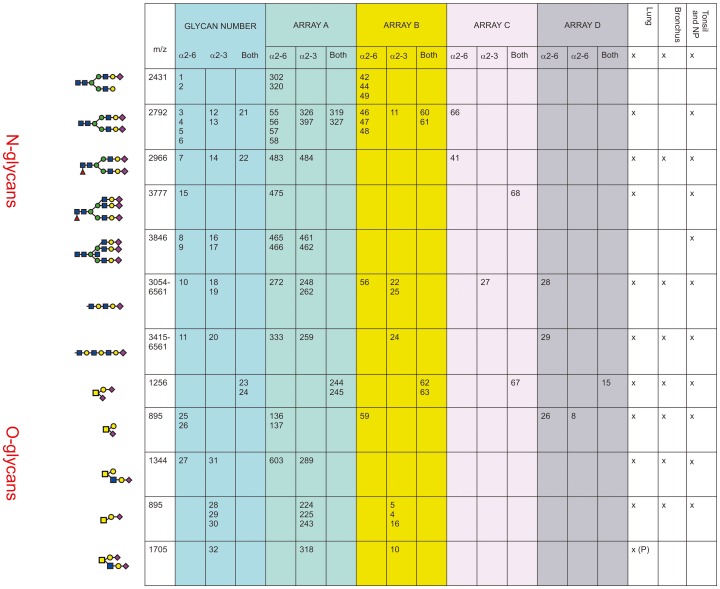
Comparison of glycan arrays for the presence of glycans identified in the bronchus and lung. The arrays compared are those from The Consortium of Functional Glycomics (CFG) (Array A Version 5); the array used by The Center for Disease Control and Prevention, (Array B), the array used by Childs and colleagues (Array C) [7], and the array used by Wong and Colleagues (Array D) [8]. The numbers in each column refer to the specific glycan number used for the respective array and the figure in the first column is the cartoon representation of each specific glycan. The linkage of the galactose to the sialic acid is identified as α2-6 and/or α2-3. The column labeled Glycan Number refers to the 32 glycans present on arrays that were identified as being present in the lung, bronchus, tonsil and nasopharynx and used in Figures 6–10. The presence or absence of these glycans in different regions of the respiratory tract is indicated by a x.

In Figure 5 the glycan composition of the available glycan arrays with the glycans present in human airways was analyzed. The glycan arrays examined were designated A–D: The Consortium of Functional Glycomics (CFG) (Array A Version 5); the array reported by The Center for Disease Control and Prevention, (Array B), the array reported by Childs and colleagues (Array C), and the array reported by Wang and Colleagues (Array D). The sialylated glycans of interest that share structural features with those indentified in the MS samples are listed in the column Glycan Number from 1–32.

Arrays A, B and C contained the α2-6 biantennary disialylated non core-fucosylated glycan (m/z 2792, i.e. Figure 5, glycans 3-6) with array A and B also containing the α2-3 variant (Figure 5, glycans 12–13) and the α2-6/α2-3 glycan (Figure 5, glycans 21–22). Arrays C and A contained the core fucosylated glycan (m/z 2966, i.e. Figure 5, glycan 7) with array A containing both the α2-3 and α2-6 glycans. These two arrays also contained the tri-antennary tri-sialylated glycan (m/z 3777, i.e. Figure 5, glycan #15) in a α2-6 configuration however it is not possible at this stage to determine if all the possible combinations of α2-3/α2-6 triantennary glycan exists in a physiological state. Only array A contained the bisecting triantennary (3846 m/z, i.e. glycan 8, 9, 16, 17). Extended sialylated LacNacs were present on glycans at m/z 3054, 3299, 3415, 3503, 3865, 3953, 4314, 4402, 4763, 4851, 5213, 5662, 6112 and 6561 some of which were present on all arrays (Glycans 10, 11, 18, 19, 20). All arrays contained the sialylated core-1 O-glycans at m/z 895 and 1256, which were glycans 23-26 and 28-30. Array A identified the core-2 glycans (27, 31 and 32). We then determined to what extent these arrays contained glycans present in human lung and bronchial samples. Figure 1 demonstrated over 20 different sialylated N-glycan molecular ions, and 4 different sialylated O-glycan molecular ions were observed in the glycomic analysis. However, it should be noted that due to micro-heterogeneity (for example varying lengths of LacNAc extensions and positions of sialylation on multi-antennary glycans) that the actual number of potential sialylated structures is much larger. 12 of these potential glycans were present on the combined arrays (Figure 5), however none of the arrays represented all the glycans present in the human airways. Array's A and B contained the largest number of potential human airways sialylated structures compared to arrays C and D.

### The ex-vivo cultures of the lung had a wider range of infections with influenza viruses than the bronchus

We compared the binding of different viruses on glycan array A with the ability of these viruses to replicate in *ex vivo* cultures of human bronchus and lung. We are cognizant that while efficient replication in these *ex vivo* cultures implies efficient binding to the virus cell receptor, the converse is not always true. Thus, viruses that bind and are internalized by receptor mediated pathogenesis may not always replicate productively in the epithelium [12]. A total of 113 bronchial and 185 lung samples were infected between 2008 and 2012 (Table 1). We defined a ≥1 log increase in TCID_50_ as evidence of productive replication and an increase of between 0.5–1 log increase as evidence of partial replication in the bronchus and lung. We have indicated the overall percentage of *ex vivo* cultures with productive and non productive replication of different subtypes and characterized viruses that had a high (>80%) infection rate and those with a low (<20%) infection rate (Table 1). As bronchial tissue samples were more scarce than lung samples, we were not able to have an identical number of experiments done for all viruses. Apart from 2 swine viruses (A/Swine/Hong Kong/4167/1999 and A/Swine/Hong Kong/915/2004) all viruses tested showed replication in the lung, with an efficiency of replicating ranging from 25–100%. Sialidase treatment abolished infection in the H1N1pdm and H5N1 infected tissues [11], [13]. In the bronchial explants, apart from A/Swine/Hong Kong/NS29/2009 and the 2 H5N1 viruses, replication ranged from 21–100%.

**Table 1:** Viruses used and their replication in *ex-vivo* tissues.

VIRUS					EX VIVO REPLICATION			
Virus Name	Origin	Year	Subtype	Abbreviation	n	% replicationin bronchus	n	% replication in lung
A/HongKong/54/1998	Human	1998	H1N1	HK98/H1N1	16	69	54	78
A/Oklahoma/447/2008	Human	2008	H1N1	OK08/H1N1	16	69	27	89
A/Oklahoma/1137/2009	Human	2009	H1N1	OK09/H1N1	0	N/A	3	100
A/HongKong/415742/2009	Human	2009	H1N1pdm	HK09/H1N1pdm	19	90	24	78
A/Oklahoma/3052/2009	Human	2009	H1N1pdm	OK09/H1N1pdm	2	100	10	70
A/Oklahoma/3003/1996	Human	1996	H3N2	3003/H3N2	0	N/A	9	78
A/Oklahoma/5098/1996	Human	1996	H3N2	5098/H3N2	0	N/A	4	50
A/Oklahoma/323/2003	Human	2005	H3N2	323/H3N2	0	N/A	6	67
A/Oklahoma/1992/2005	Human	2005	H3N2	1992/H3N2	13	86	13	54
A/Oklahoma/309/2006	Human	2006	H3N2	309/H3N2	0	N/A	5	20
A/Oklahoma/483/2008	Human	2008	H3N2	483/H3N2	0	N/A	7	86
A/Oklahoma/5386/2010	Human	2010	H3N2	5386/H3N2	9	67	9	33
A/Oklahoma/5342/2010	Human	2010	H3N2	5342/H3N2	8	33	8	25
A/HongKong/483/1997	Human	1997	H5N1	HK97/H5N1	1	0	0	N/A
A/Vietnam/3046/2004	Human	2004	H5N1	VN04/H5N1	1	0	17	65
A/Netherland/219/2003	Human	2003	H7N7	NL219/H7N7	0	N/A	5	100
A/Netherland/33/2003	Human	2003	H7N7	NL33/H7N7	0	N/A	5	100
A/HongKong/1073/1999	Human	1999	H9N2	99/H9N2	5	40	5	100
A/HongKong/2108/2003	Human	2003	H9N2	03/H9N2	5	40	5	60
A/HongKong/464419/2009	Human	2009	H9N2	09/H9N2	5	80	5	100
A/Swine/HongKong/4167/1999	Swine	1999	H1N1	swHK99/H1N1	9	25	3	0
A/Swine/Gent/112/2007	Swine	2007	H1N1	swG07/H1N1	3	75	3	100
A/Swine/HongKong/1559/2008	Swine	2008	H1N1	sw1559/H1N1	7	25	4	75
A/Swine/HongKong/NS29/2009	Swine	2009	H1N1	swNS29/H1N1	5	0	17	41
A/Swine/Hong Kong/201/2010	Swine	2010	H1N1	sw201/H1N1	13	21	5	75
A/Swine/Arkansas/2976/2002	Swine	2002	H1N2	swAR02/H1N2	7	33	10	70
A/Swine/HongKong/915/2004	Swine	2004	H1N2	sw915/H1N2	5	64	8	0
A/Duck/Bavaria/1/1977	Duck	1977	H1N1	dk/H1N1	3	33	7	57
A/northern pintail/HongKong/MP5883/2004	Northern Pintail	2004	H5N8	np/H5N8	2	50	18	55
A/Chicken/Netherlands/1/2003	Chicken	2003	H7N7	ck/H7N7	0	N/A	5	100
A/Quail/HongKong/G1/1997	Quail	1997	H9N2	qu/H9N2	9	78	14	79
A/Chicken/HongKong/Y280/1997	Chicken	1997	H9N2	ck/H9N2	5	80	13	69

The seasonal H1 and H3, avian H1, H5,H7 and H9, and swine viruses are listed. The % of productive replication in *ex vivo* cultures of bronchus and lung is shown together with the number of samples used for each experiment. Productive replication was defined as >0.5 log replication as determined by TCID_50_.

### Glycan binding of H1N1, H3N2, H1N1pdm, swine, avian and H9N2 influenza A viruses using array A (CFG glycan array) platform was not predictive of virus replication

**Figure 6:**
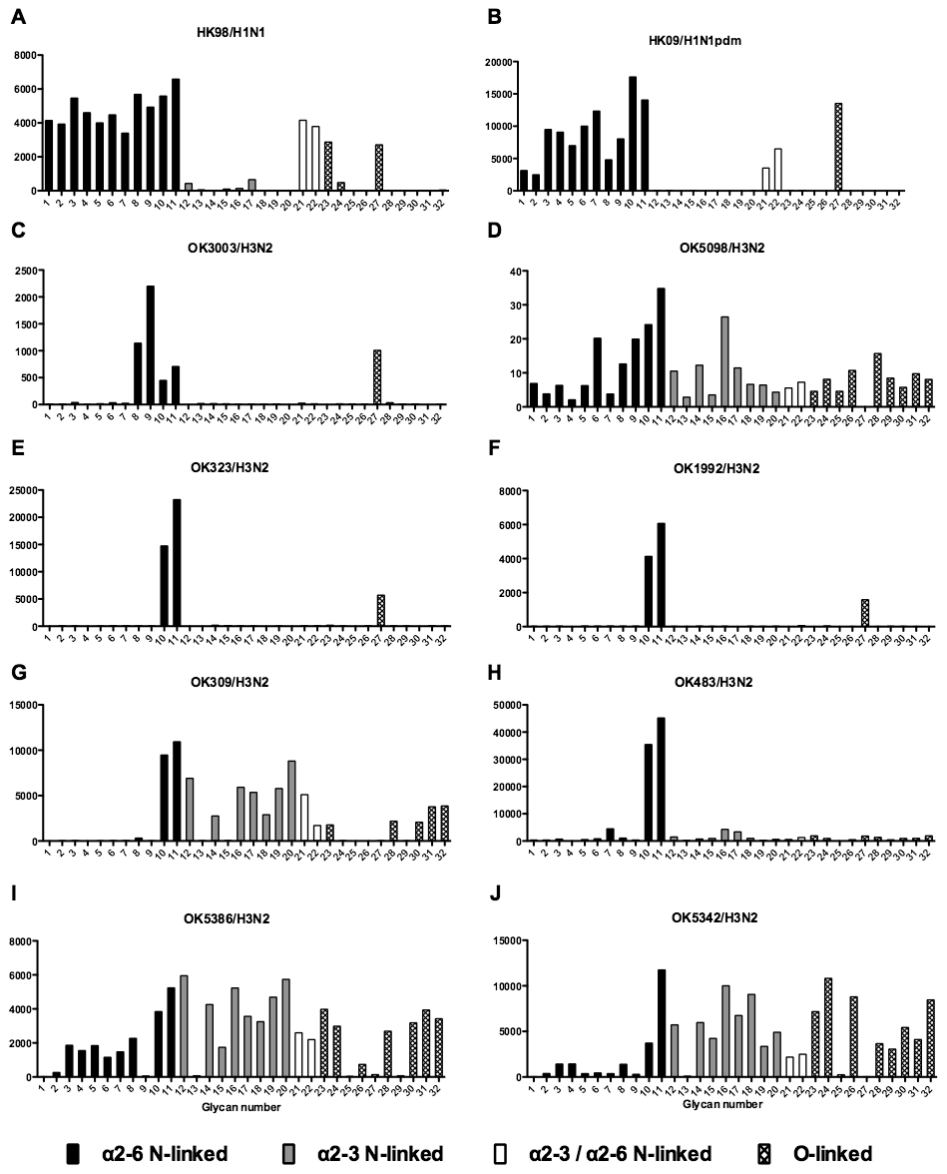
Glycan array analysis of seasonal and pandemic H1 viruses. Different types of sialoglycans on the array (x-axis) present in the human respiratory tract are indicated by numbers referred to in Figure 5 and shading/hatch refers to whether the glycans are N-linked or O-linked. Vertical bars denote mean binding signal (fluorescence intensity). A is a seasonal H1N1 virus, B is a H1N1pdm virus and C-J are seasonal H3N2 viruses.

**Figure 7:**
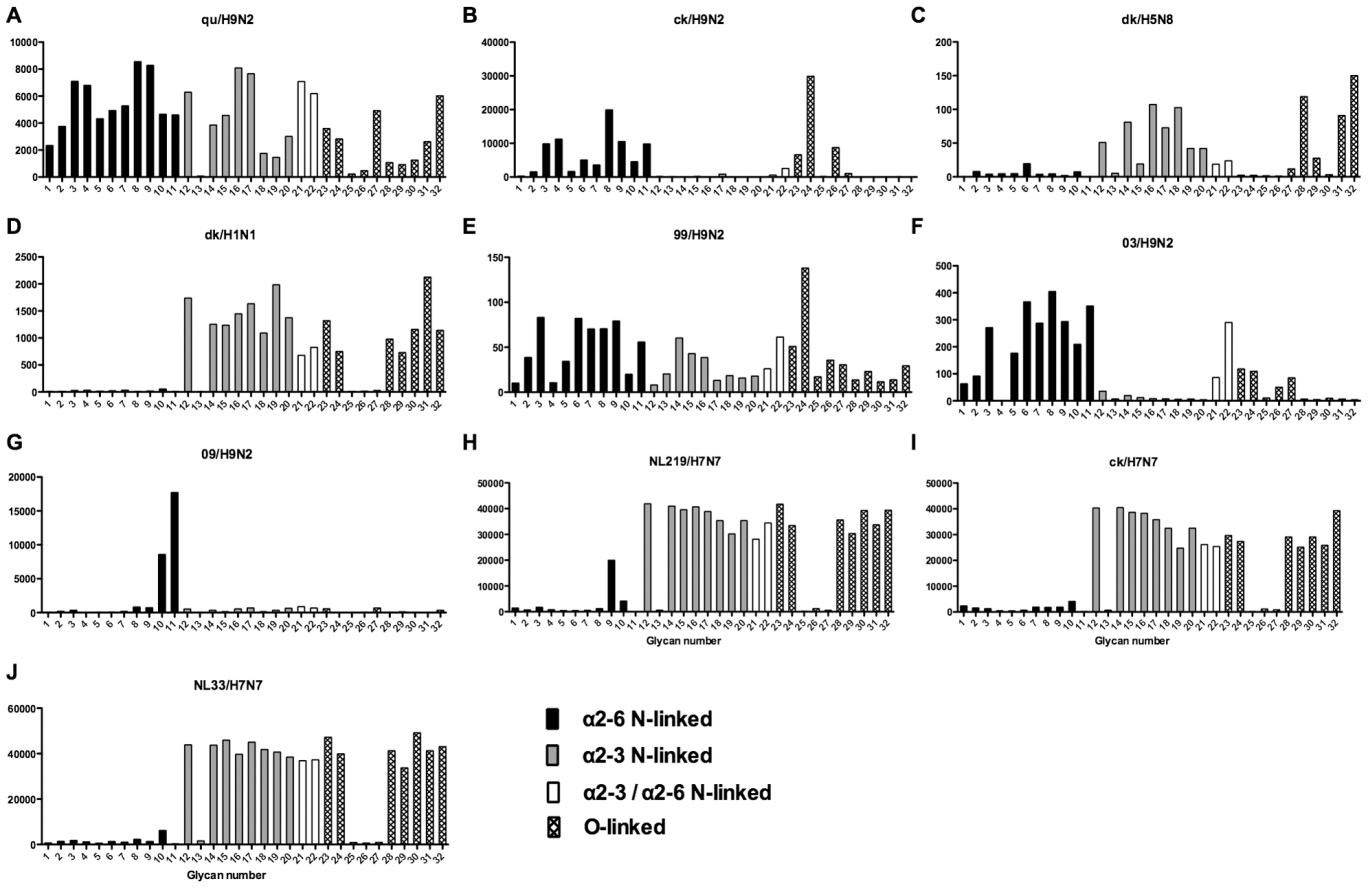
Glycan array analysis of avian-origin and H9N2 viruses. Different types of sialoglycans on the array (x-axis) present in the human respiratory tract are indicated by numbers referred to in Figure 5 and shading/hatch refers to whether the glycans are N-linked or O-linked. Vertical bars denote mean binding signal (fluorescence intensity). A and B are avian isolated H9N2 viruses, C is an avian H5N8 virus, D is duck isolated H1N1, E–G are H9N2 viruses isolated from humans, and H–J are H7N7 viruses.

**Figure 8:**
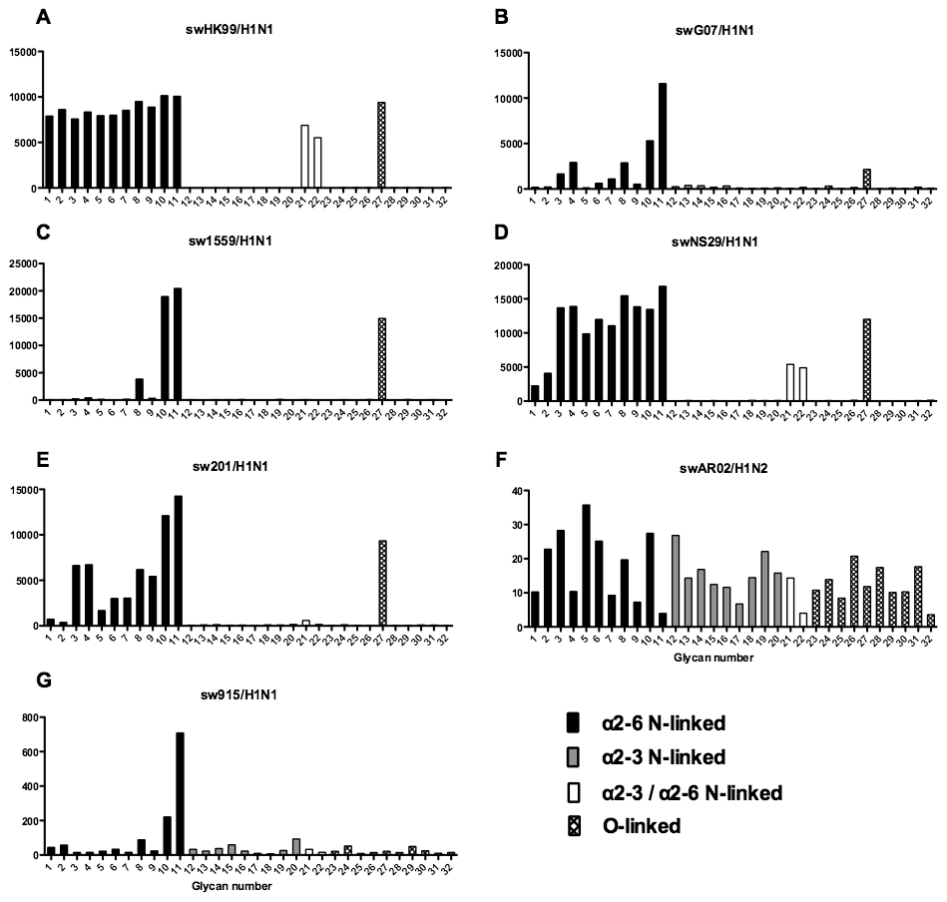
Glycan array analysis of swine H1 viruses. Different types of sialoglycans on the array (x-axis) present in the human respiratory tract are indicated by numbers referred to in Figure 5 and shading/hatch refers to whether the glycans are N-linked or O-linked. Vertical bars denote mean binding signal (fluorescence intensity). A–E and G are swine isolated H1N1 viruses and F is a swine isolated H1N2 virus.

As array A was found to have the greatest coverage of the diverse glycans found on the human airways, we examined the binding of the human, avian and swine influenza A viruses utilized in the preceding section to the 32 glycans present on the array and identified by MS analysis in human respiratory tissues. The full glycan array profiles are available on the CFG website (http://www.functionalglycomics.org/). It should be noted that 8–10 of the potential binding glycan structures were not present in previous array results (OK/447/08, OK 1137/09 and 3052/09). The results are shown in Figures 6–8. In general all human influenza virus strains of seasonal H1N1 subtype bound a wide range of α2-6 N-glycans, α2-3/α2-6 biantennary N-glycans and limited α2-3 N-glycans (Figure 6A–B). HK98/H1N1 and HK09/H1N1 also had limited binding to O-glycans.The H3N2 viruses (Figure 6C–J) showed consistent binding to the extended α2-6 biantennary LacNAc (Glycans 10 and 11) but varied binding to α2-3N-glycans and O-glycans. The avian H9N2 viruses also showed variation in binding (Figure 7A,B) and the 2 duck viruses (Figure 7C,D) were mainly α2-3N-glycan binding and O-glycan binding. The 3 human isolated H9N2 viruses (Figure 7E–G) bound mainly α2-6 N-glycans apart from the 99/H9N2 which showed increased O-glycan binding. As published previously the H7N7 viruses (Figure 7H-J) were mainly α2-3 N-glycan and O-glycan binding, however the NL219/H7N7 did show slight α2-6 binding. The swine viruses (Figure 8) were mainly α2-6 N-glycan binding with a single core 2 O-glycan binding identified (Figure 8A–G). An exception was the swAR02/H1N2 which had a low binding across involving both α2-3 and α2-6 glycans (Figure 8F).

**Figure 9:**
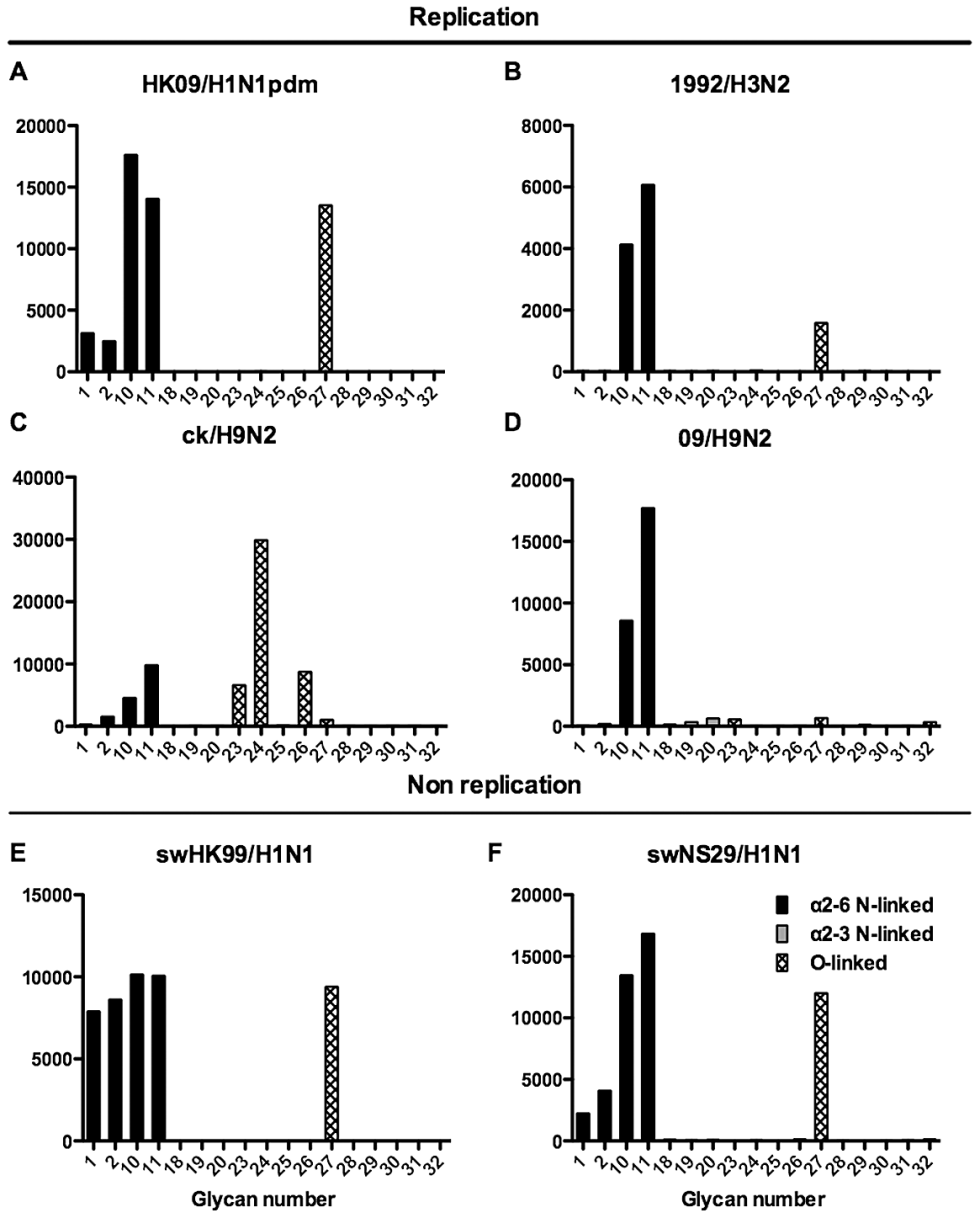
Glycan array analysis of viruses that showed no replication or replication in the bronchial explants. Binding signal of viruses that have a high degree of replication (HK09/H1N1pdm, OK09/H1N1pdm, 1992/H3N2, 09/H9N2 and ck/H9N2) and those that have a low degree of replication (swHK99/H1N1 and swNS29/H1N1) in the human ex vivo bronchus. Different types of sialoglycans on the array (x-axis) present in the human respiratory tract are indicated by numbers referred to in Figure 5 and shading/hatch refers to whether the glycans are N-linked or O-linked. Vertical bars denote mean binding signal (fluorescence intensity). A is the pandemic H1N1 virus isolated in Hong Kong, B is H3N2 human virus, C is an avian H9N2 virus and D is a human H9N2 virus. E and F are swine isolated H1N1 viruses.

**Figure 10:**
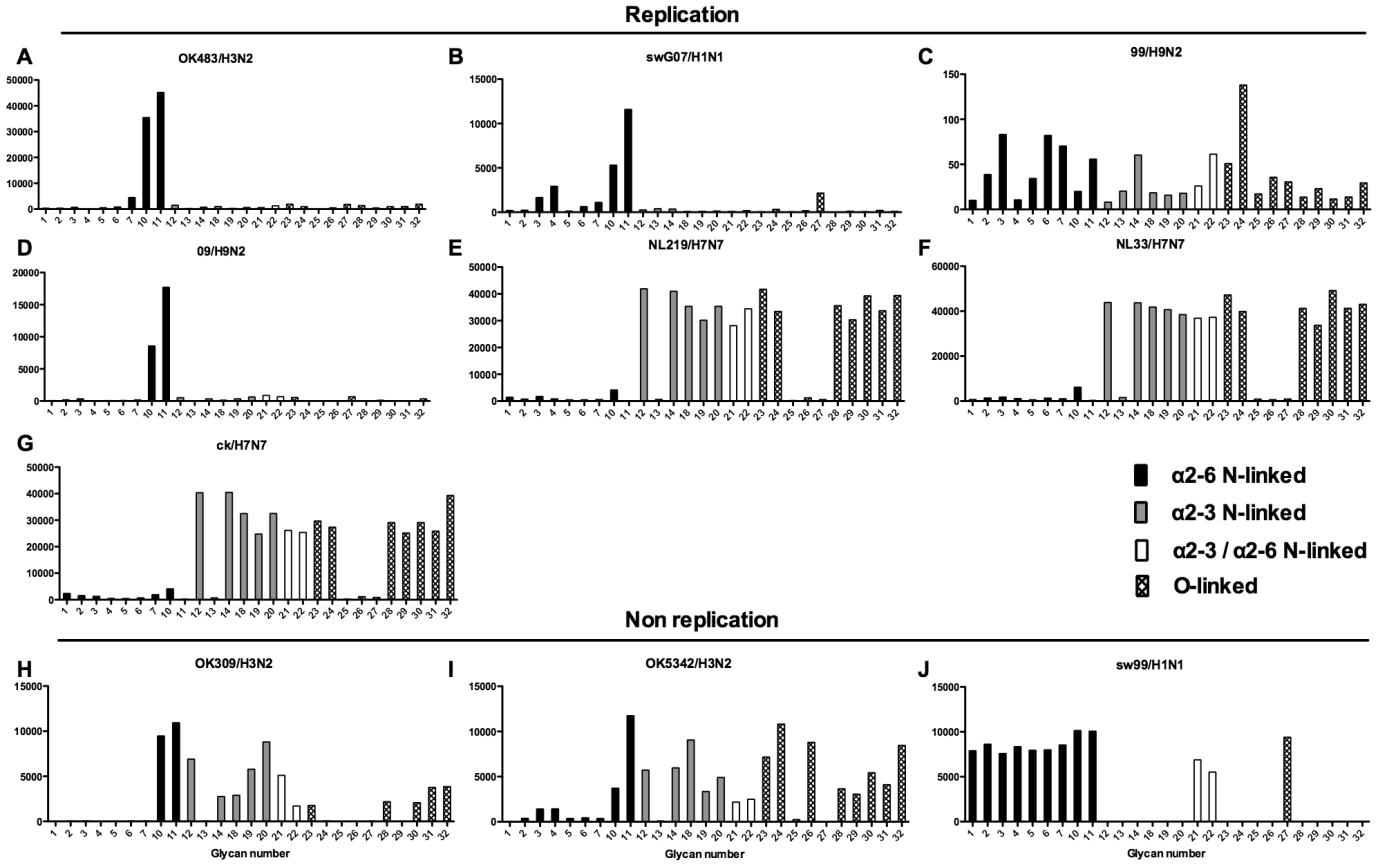
Glycan array analysis of viruses that showed no replication or replication in the lung explants. Binding signal of viruses that have a high degree of replication (OK483/H3N2, swG07/H1N1, 99/H9N2, 09/H9N2 NL219/H7N7, NL33/H7N7,and ck/H7N7 and those that have a low degree of replication (309/H3N2, OK5342/H3N2 and swHK99/H1N1) in the human ex vivo lung. Different types of sialoglycans on the array (x-axis) present in the human respiratory tract are indicated by numbers referred to in Figure 5 and shading/hatch refers to whether the glycans are N-linked or O-linked. Vertical bars denote mean binding signal (fluorescence intensity). A is a human H3N2 virus, B is a swine isolated H1N1 virus, C and D are human isolated H9N2 viruses and E–G are H7N7 viruses. H and I are human H3N2 viruses and J is a swine H1N1 isolated virus.

As binding is the first step in the process leading to productive replication, we finally sought to investigate the array data of viruses that showed >80% of partial or productive replication in the bronchus and lung and compare these profiles with viruses that had a low (<20%) rate of infection to see if there were binding profiles present on the current array that would correlate with productive replication. For the bronchus we selected HK09/H1N1pdm, 1992/H3N2, 09/H9N2 and ck/H9N2 as these 4 viruses had a high level of replication. The 2 low replicating viruses were swHK99/H1N1 and swNS29/H1N1. For the lung we selected OK483/H3N2, swG07/H1N1, 99/H9N2, 09/H9N2, NL219/H7N7, ck/H7N7 and NL33/H7N7 as efficient replicators and OK309/H3N2, OK5342/H3N2 and sw99/H1N1 as low replicators. The comparative array data for the bronchus and lung is shown in Figures 9 and 10 respectively. For the bronchus, the only α2-6 binding common glycans for the high replicating viruses were 10, and 11 (Figure 9A–D) which are α2-6 extended LacNAcs but these had a high degree of binding to non-replicating viruses (Figure 9E-F). No common high binding glycans were identified that distinguished replicating viruses (Figure 10A–G) from non-replicating viruses (Figure 10H–J) in the lung.

## Discussion

### Glycomic analysis demonstrates a wide spectrum of sialylated N and O-linked glycans in the lung and bronchus which can bind many respiratory viruses, such as parainfluenza and influenza

There were three aims in this study: to obtain a comprehensive analysis of the sialylated glycans present in the normal lung, to determine if the available arrays were representative of the true glycans present in the human respiratory tract, and finally to investigate if there was a regional difference in influenza virus infection between the bronchus and lung, and if so, whether this could be explained by a different glycan composition. With respect to the first aim we found that was a wide range of sialylated N and O-glycans present in the lung and bronchus and that there were regional differences between these two locations with the lung having a higher degree of sialylation. Sialidase digestion showed that there was both α2-6 and α2-3 linked Sia present in both these locations.

The main N-glycans present were complex type multiantennary with LacNAc extentions (Figure 1) and Core 1 and 2 O-glycans (Figure 4). A striking feature of both the lung and bronchus data was the presence of large complex N-glycans with sialylated poly-LacNAc chains. Such glycans have previously been identified in swine and human respiratory epithelial cells and have been implicated in adaptation and infection of influenza viruses in these hosts [14], [15]. Our definitive evidence for such structures being present in the human respiratory tract add credence to such arguments. Greater insight of the role of such glycans is also coming from the development of a custom glycan microarray of sialylated poly-LacNAc containing N- and O- glycans as well as linear terminal fragments [16]. As bacteria such as Pseudomonas [17],and other respiratory viruses apart from influenza, such as parainfluenza utilize Sia in binding (reviewed in [18]), our glycan data will be beneficial in determining the potential tropism of these pathogens within the human respiratory tract.

A large body of glycan structural data has emerged from analysis of secreted mucins from cystic fibrosis and chronic bronchitis patients [19], [20], [21], [22] and analysis of neutral low molecular weight N-glycans isolated from paraffin-embedded archival tissue samples of normal and cancer lung tissue [22], however rigorous structural analysis has hitherto not been applied to normal human respiratory tract tissues. To date the limited data on the actual glycan composition of the respiratory tract has been obtained from cultured bronchial epithelial cells [15]. These cells in culture may not ideally represent glycan profiles of actual respiratory tract tissues. The authors in that study did not detect the biantennary non-core fucosylated mono-sialylated glycan at 2431 m/z and found more abundant tri-fucosylated glycans at 2859 m/z (non-permethylated). Furthermore they appeared to detect Gal-Gal linkages at m/z 2894.9 and 3057 which were not identified in our samples. The differences can be explained by the source of tissues and changes that might be induced by culture conditions [23]. O-glycan profiles were not mentioned. Though Chandrasekaran and colleagues did not find evidence of α2-3 terminated N-glycans, our previously published lectin binding results [24] and current sialidase treatment of bronchial tissues did find evidence of these glycans. Similar to previously published lectin binding experiments [24] the paediatric lungs showed more α2-3 terminated glycans. This could possibly explain the higher fatality rates of children infected with H5N1 [25].

### The current glycan arrays do not represent the full spectrum of N-glycans present in the human lung and bronchus

Given that we identified a broad range of glycans in lung and bronchial tissues, the second aim of the project was to identify which of these glycans were present on the available glycan arrays with the findings presented in Figure 5. We found that while the O-glycans are well represented on the 4 platforms, the greater complexity and number of N-glycans meant the degree of representation for N-glycans is smaller, in particular those α2-3 extended biantennary glycans which are more challenging to produce than α2-6 ones. As Figure 5 demonstrates, the full array for the CFG(Array A) is the most comprehensive, and this has recently been confirmed by chemoinformatic tools [26]. From the full 611 array we used the MS results from Figure 1 to select 32 sialylated glycans that were present on the array, and present in the human lung and bronchus to determine if this select 32 array would be sufficient to predict infection of ex-vivo tissues.

### Glycan binding profiles of human, swine and avian viruses demonstrates a wide variation in binding to a range of α2-3 and α2-6 glycans

The glycan binding profiles of influenza viruses selected for ex vivo infection is listed in Figures 6–8. This showed that categorizing virus groups such as H1N1 or H3N2 as “α2-6” or “α2-3” was too simplistic and that individual strains showed wide variation in receptor preference. Thus the current paradigm of categorizing viruses as only “α2-6” or “α2-3” binding to explain the tropism of influenza viruses is thus an over simplification that may lead to false conclusions.

The H7N7 viruses analyzed were mainly α2-3 binding (Figure 7H–J) but the A/NL/219/2003 showed a single α2-6 peak corresponding to a trisialylated triantennary bisecting glycan (Figure 7H glycan 9). This structure may contribute to the ”weak” α2-6 haemagglutination found by one group of investigators [27] in resialylated turkey red blood cells, and for the increased replication in certain cell lines. Since all 3 H7N7 viruses replicated in our human lung *ex vivo* tissues this increased α2-6 binding cannot explain the different tropism seen clinically. This glycan was not identified as a major species in the lung or bronchus by mass spectrometry and was not present on the array used by the CDC in their analysis of H7N7 viruses [28]. Munster and colleagues found different patterns of viral attachment in lung samples between A/NL/219/03 (H7N7) and A/NL/33 (H7N7) [29] but both these viruses appeared to replicate in our human *ex vivo* samples (Table 1)

### The currently available glycan arrays at this stage are not able to predict infection of human respiratory tract tissues

Finally, to determine if the select 32 glycan array is able to predict productive replication in the human bronchus and lung, we selected viruses which had more than a 0.5 log increase in TCID_50_, and those that showed no evidence of replication, and examined whether there was any consistent glycan profiles in the 32 sialylated glycan array that would predict infection. The results, as shown in Figures 9 and 10 suggested that there appeared to be some common high binding glycans – in particular numbers 10 and 11 which are extended LacNAcs. However these glycans had a high binding for viruses that did not replicate in the bronchus (e.g. swHK99/H1N1 and swNS29/H1N1) which implies either that there may be other glycan structures that are involved in replication which are not present on the current array, or that other gene components of swine viruses inhibit successful productive replication in this tissue. It is possible that the extended LacNAc glycans (glycan numbers 10 and 11) may exert their preferential binding owing to their greater length. Due to limited amounts of human samples we did not attempt to identify the glycolipids present in the lung and bronchus, but from the array data it appears that apart from the dk/H1N1 and Np/H5N8 none of the seasonal viruses had any strong binding to gangliosides such as GD1a (data not shown). As there was no clear link between binding and replication, experiments using labeled virus binding assays, though informative from a morphological aspect, may not be representative of the capacity for effective virus infection [23], [24].

### Alveolar tropism by influenza viruses appears to involve both α2-3 and α2-6 glycans

Our findings identifying α2-6 as well as α2-3 terminated glycans in the lung by mass spectrometric analysis highlights that alveolar tropism of influenza viruses is not determined solely by the ability to bind α2-3 glycans, but that α2-6 glycans can play a part. Though human surfactant has not been analyzed in detail, porcine surfactant has both α2-3 and α2-6 glycans which are able to interact with influenza viruses [30] and such ability to evade such factors may be important for successful replication of a virus within the alveolar spaces.

The H1N1pdm virus in 2009 was largely an upper airways disease but was able to replicate in the lower, as well as upper respiratory tract as seen both in clinical patients [31] as well as *ex vivo* cultures [9]. The glycan array results (Figure 6) as well as previously published findings [32], [33] show that H1N1pdm is α2-6 restricted in its binding.

Previous research suggested that the tissue tropism and pathogenesis of H5N1 was due to the targeting of the virus to the alveolar epithelium where lectin histochemistry indicated there was an abundance of α2-3 linked receptors which this avian virus binds to. The lack of transmission between humans was attributed to the lack of α2-3 linked receptors (as determined by lectin binding studies) in the upper respiratory tract which was believed to preclude virus replication, thus making mammalian transmission unlikely to occur [3]. However, our findings of infection of bronchi by predominantly α2-3 binding viruses such as dk/H1N1 and np/H5N8 as well as the identification of α2-3 sialylated glycans in this location suggest this paradigm might need revision. Furthermore, our MS analysis, and *ex vivo* cultures demonstrated that “pure” α2-6 binding such as OK483/H3N2, 09/H9N2 and swG07/H1N1 viruses can replicate in the lungs (Figure 10). In addition, recent studies on H5N1 replication and transmission in ferrets have demonstrated replication of viruses containing the H5N1 haemagglutinin in the upper respiratory tract [34].

### Future glycan arrays should incorporate the common glycans present in the bronchus and lung

In conclusion we have shown data demonstrating a wide diversity of α2-6 and α2-3 N and O-glycans in the human adult and paediatric respiratory tract and correlated this with existing glycan array binding data for circulating human, avian and swine viruses. Though some of the currently available glycan arrays have some of these glycans represented, none of them are comprehensive in having all the key glycans present in human airways and thus binding profiles were not predictive of replication in the bronchus or lung. Updating glycan arrays to express the full spectrum of glycans present in human airways is a priority in order to understand the biology of influenza virus transmission and pathogenesis and for risk-assessing animal viruses for pandemic threat.

## Materials and Methods

### Influenza virus preparation

The viruses used in the present study included highly pathogenic H5N1 virus: A/VN/3046/04 (H5N1) and low pathogenic avian influenza viruses: A/Northernpintail/HK/MP5883/2004 (H5N8), A/Duck/Bavaria/1/1977 (H1N1), A/Quail/HK/G1/97 (H9N2) and A/Chicken/HK/Y280/97 (H9N2), and seasonal and swine influenza virus H1N1, H1N2 and H3N2 (Table 1). Virus stocks were propagated in Madin-Darby Canine Kidney (MDCK) cells. The stock was titrated as previously described [35]. Replication competence and kinetics of these viruses was tested in an MDCK culture.

### Mass spectrometric analysis

Three lungs and two bronchi from adult patients and two lungs and one bronchi from 1 paediatric patient (<3 years) were sampled from normal surgical samples sent for pathology examination. The collection of tissues was approved by an Institutional Review Board. The lung and bronchial N-glycans were prepared for mass spectrometric analysis as previously described [36]. Briefly, the tissues were homogenized and sonicated in a homogenization buffer of 1% CHAPS (v/v) in 25 mM Tris, 150 mM sodium chloride (NaCl), 5 mM EDTA in water, pH 7.4 and subsequently dialyzed against a 50 mM Ammonium bicarbonate (Ambic) buffer. The samples were reduced, carboxymethylated and digested with Trypsin (EC 3.4.21.4) Sigma). The N-glycans were released by digestion with PNGase F (EC 3.5.1.52; Roche Molecular Biochemicals) and purified by reverse-phase C18 Sep-Pak (Waters) chromatography. Sialidase cleavage was performed with Sialidase S (recombinant from *Streptococcus pneumoniae* expressed in *E. coli*, Glyco, 170 mU) and Sialidase A (recombinant from *Arthrobacter ureafaciens* expressed in *E. coli*, Glyco, 170 mU), in 50 mM Sodium acetate, pH 5.5. The digest samples were lyophilized, permethylated and purified by C18 Sep-Pak (Waters). Matrix assisted laser desorbtion ionization-time of flight (MALDI-TOF) data were acquired on a Voyager-DE STR mass spectrometer (PerSeptive Biosystems, Framingham, MA) in the reflectron positive mode with delayed extraction. Permethylated samples were dissolved in 10 µl methanol and 1 µl of dissolved sample was premixed with 1 µl of matrix (20 mg/ml 2,5-dihydroxybenzoic acid [DHB] in 70% [vol/vol] aqueous methanol before being loaded onto the sample plate. MALDI-TOF/TOF experiments were performed on a 4800 Proteomics Analyzer (Applied Biosystems, Framingham MA) operated in the reflectron positive ion mode.

### Glycan array

The influenza viruses isolated from Hong Kong, listed in Table 1, were propagated and purified using ultra-centrifugation. The concentrated viruses were inactivated by paraformaldehyde and labeled with Alexa 488 according to previously published methodology [37]. Analysis was performed by Core H of the Consortium for Functional Glycomics at an initial dilution of 1∶20 with increasing concentrations to saturation on the same slide. Other recent H3N2 viruses (OK) were purified by sucrose density centrifugation and labeled with Alexa488. The glycan array binding data from these viruses was obtained from the Consortium for Functional Glycomics website (http://www.functionalglycomics.org/).

### *Ex vivo* tissue cultures and influenza virus infection

*Ex vivo* culture of human bronchus and lungs was performed as previously described [38], [39] and the number of experiments that used each virus strain were listed in Table 1. The collection of lung and upper respiratory tract tissues was approved by the University of Hong Kong/Hospital Authority Hong Kong West Cluster (HKU/HA HKW IRB) with written informed consent provided by study participants and/or their legal guardians. The tissues were obtained from patients undergoing surgical biopsy or resection for pathological diagnosis, and consent had been given for the use of these tissues for influenza research. The normal tissues that were used were surplus to routine diagnosis. 3 samples were pre-treated with a bacterial sialidase before infection with H5N1 and H1N1pdm [11], [13]. In brief, fresh bronchial and lung tissues were infected with influenza virus with a titer of 10^6^ 50% tissue culture infectious doses (TCID_50_)/ml, a titer similar to that used previously [38], [39], [40] for 1 h at 37°C and washed with 5 ml of warm 1× phosphate-buffered saline for three times to remove unbound virus. The bronchial mucosa was placed on a surgical sponge with its apical epithelial surface upwards in a 12 well plate with 1.5 ml of culture medium at 37°C while the lung parenchyma was placed into the 12 well plate directly with 1.5 ml of culture medium. To determine productive viral replication from the infected biopsy specimens, supernatants of the infected cultures were collected at 1, 24, 48 and 72h post infection (hpi) and stored at −80°C for virus titration using TCID_50_ assay in MDCK cells as described previously [38], . The increasing virus titers along the time course provided evidence of productive virus replication.

## Supporting Information

Text S1 **Supplementary figures and tables.**
(PDF)

Text S2 **Supplementary table of glycan composition with relative abundance.**
(XLS)
