# Supplementary material for: Glycomic Analysis of Human Respiratory Tract Tissues and Correlation with Influenza Virus Infection
Source: PLoS Pathog. 2013 Mar 14;9(3):e1003223. doi: 10.1371/journal.ppat.1003223 (PMC3597497; doi:10.1371/journal.ppat.1003223)

SUPPLEMENTARY FIGURE LEGENDS

Supplementary Figure S2: MALDI-TOF/TOF MS/MS spectrum of the permethylated N-glycan at m/z 4312, derived from human lung. Assignments of the key fragment ions generated are shown.

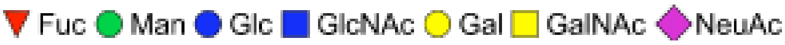

Supplementary Figure S3: MALDI-TOF/TOF MS/MS spectrum of the permethylated N-glycan at m/z 3054, derived from human bronchus. Assignments of the key fragment ions generated are shown.

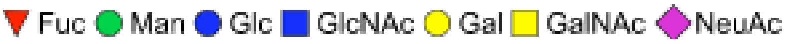

Supplementary Figure S4: N-glycan profile of human nasopharynx. N-glyan profiles of the nasopharynx were obtained from the 50% MeCN fraction from a C18 Sep-Pak column ("Experimental Procedures"). Annotated structures are according to the Consortium for Functional Glycomics guidelines. All molecular ions are [M+Na]+. Putative structures are based on composition, tandem MS, and the biosynthetic knowledge. Due to the presence of heterogeneous multiantennary structures with extended LacNAc repeats, the annotations are simplified throughout by using biantennary structures with the extensions listed in parentheses. Structures that show sugars outside a bracket have not been unequivocally defined (see supplementary Table S1).

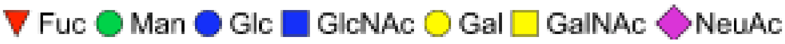

Supplementary Figure S5. Partial MALDI-TOF MS profiles of the permethylated N-linked glycans derived from paediatric human lung after digestion with sialidase S or sialidase A. Data were obtained from the 50% acetonitrile fraction and all molecular ions are present in sodiated form ($[M+Na]^+$). Sialylated species are annotated in red (see supplementary Table S1).

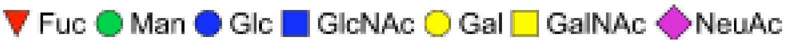

Supplementary Figure S6. Partial MALDI-TOF MS profiles of the permethylated N-linked glycans derived from peadiatric human bronchus after digestion with sialidase S or sialidase A. Data were obtained from the 50% acetonitrile fraction and all molecular ions are present in sodiated form ($[M+Na]^+$). Sialylated species are annotated in red (see supplementary Table S1).

Supplementary Figure S7 MALDI-TOF mass spectra of permethylated O-glycans of paediatric human lung and bronchus. O-Glycomic profiles of paediatric human lung (A) and paediatric human bronchus (B) were obtained from the 35% MeCN fraction from a C18 Sep-Pak column ("Experimental Procedures"). Annotated structures are according to the Consortium for Functional Glycomics guidelines. All molecular ions

are $[M+Na]^+$. Putative structures are based on composition, tandem MS, and the biosynthetic knowledge (see supplementary Table S2).

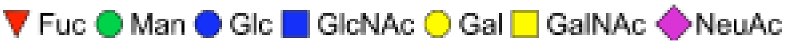

SUPPLEMENTARY TABLE LEGENDS

Supplementary Table S1: Compositional assignments, observed mass to charge ratio and relative abundance of singly charged sodiated molecular ions, $[M+Na]^+$, observed in MALDI-MS spectra of permethylated N-glycans derived from human adult lung.

Supplementary Table S2: GC-MS analyses of partially methylated alditol acetates obtained from the 50% acetonitrile fraction of PNGase F released N-glycans of human adult lung.

Supplementary Table S3: Compositional assignments, observed mass to charge ratio and relative abundance of singly charged sodiated molecular ions, $[M+Na]^+$, observed in MALDI-MS spectra of permethylated N-glycans derived from human adult bronchus.

Supplementary Table S4: GC-MS analyses of partially methylated alditol acetates obtained from the 50% acetonitrile fraction of PNGase F released N-glycans of human adult bronchus.

Supplementary Table S5: Compositional assignments, observed mass to charge ratio and relative abundance of singly charged sodiated molecular ions, $[M+Na]^+$, observed in MALDI-MS spectra of permethylated O-glycans derived from human adult lung.

Supplementary Table S6: Compositional assignments, observed mass to charge ratio and relative abundance of singly charged sodiated molecular ions, $[M+Na]^+$, observed in MALDI-MS spectra of permethylated O-glycans derived from human adult bronchus.

Supplementary Table S7: Compositional assignments, observed mass to charge ratio and relative abundance of singly charged sodiated molecular ions, $[M+Na]^+$, observed in MALDI-MS spectra of permethylated N-glycans derived from Human paediatric lung.

Supplementary Table S8: Compositional assignments, observed mass to charge ratio and relative abundance of singly charged sodiated molecular ions, $[M+Na]^+$, observed in MALDI-MS spectra of permethylated N-glycans derived from Human paediatric bronchus.

Supplementary Table S9: Compositional assignments, observed mass to charge ratio and relative abundance of singly charged sodiated molecular ions, $[M+Na]^+$, observed in MALDI-MS spectra of permethylated O-glycans derived from human paediatric lung.

Supplementary Table S10: Compositional assignments, observed mass to charge ratio and relative abundance of singly charged sodiated molecular ions, $[M+Na]^+$, observed in MALDI-MS spectra of permethylated O-glycans derived from human paediatric bronchus.

SUPPLEMENARY FIGURE 1

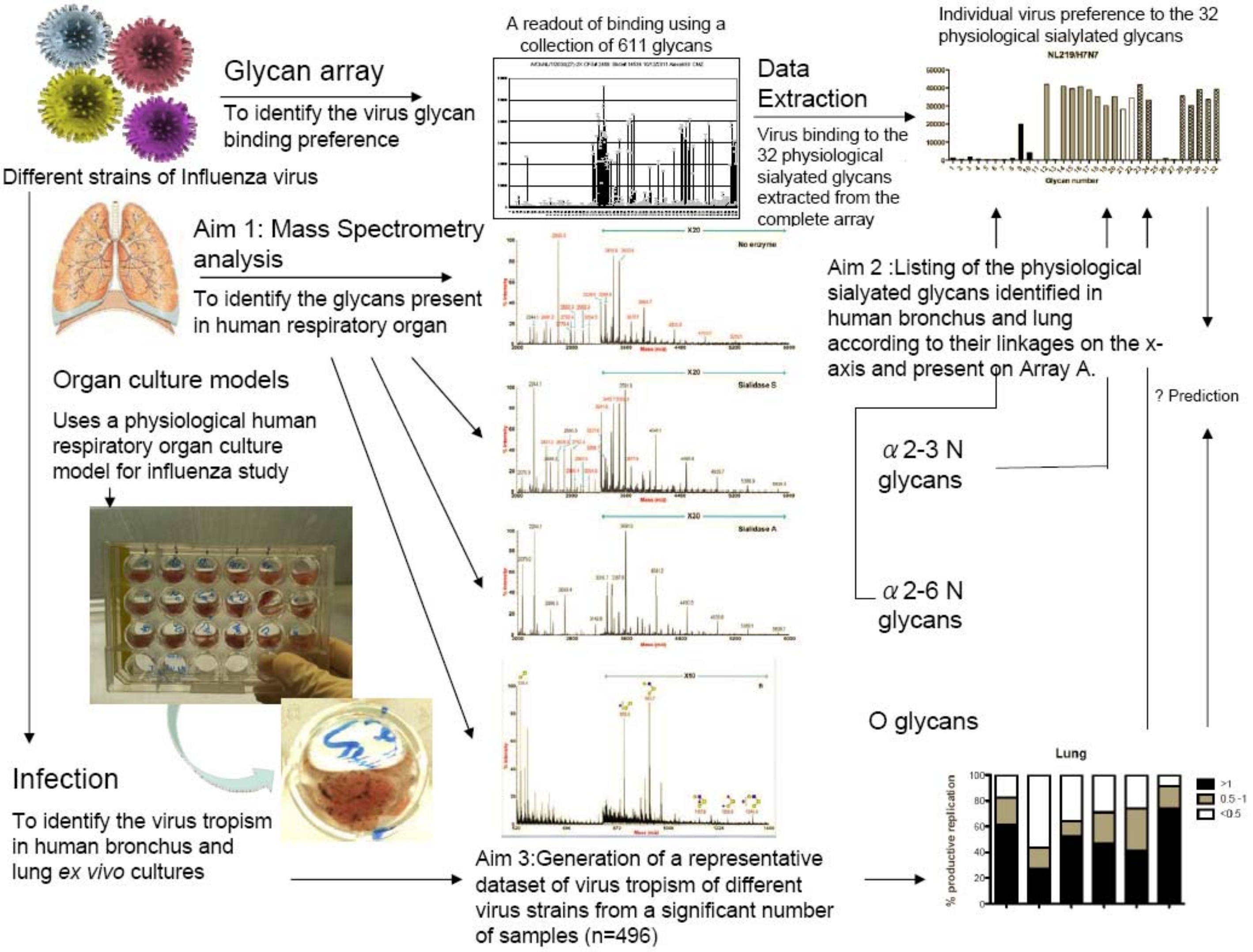

SUPPLEMENTARY FIGURE 2

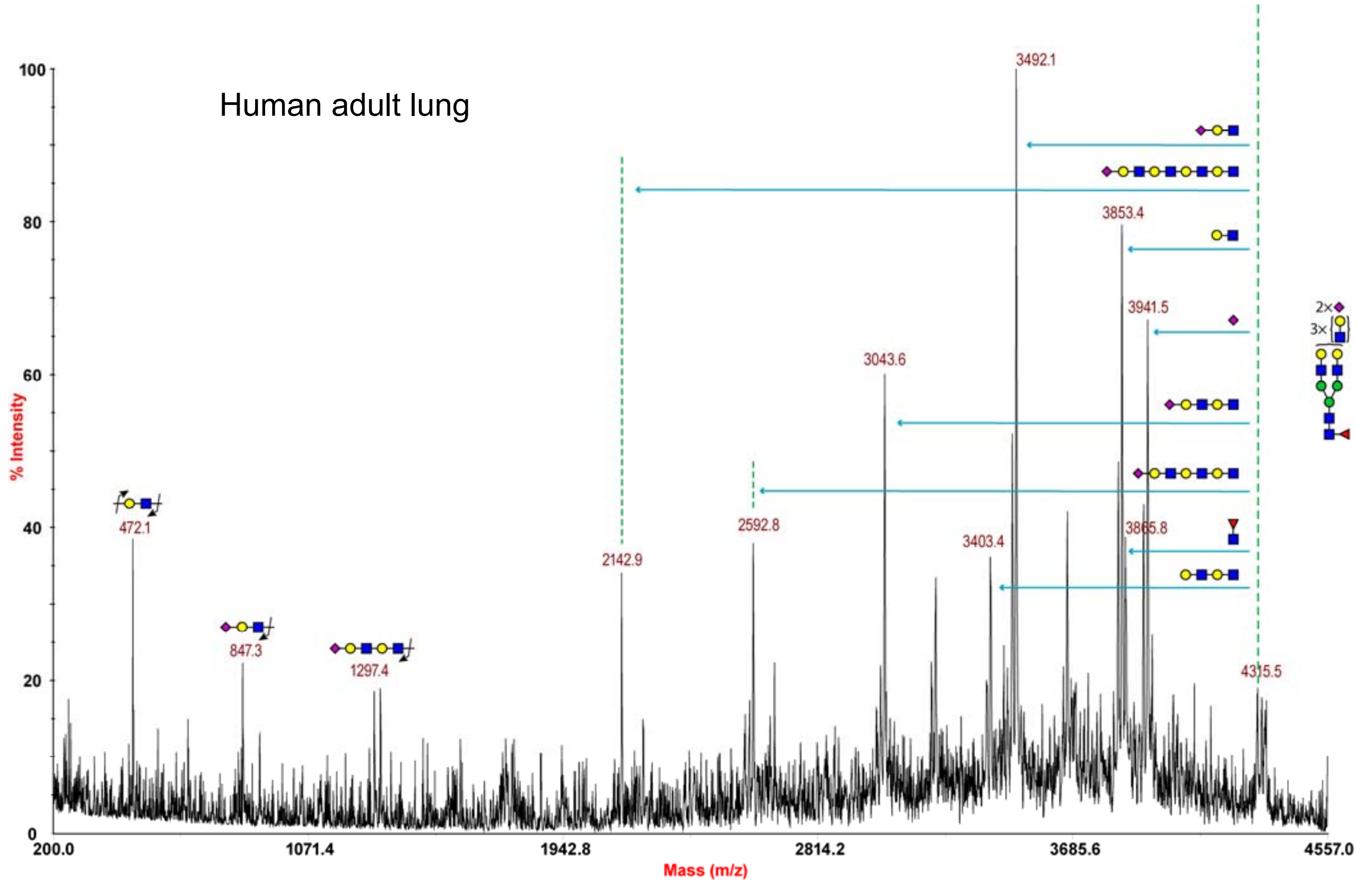

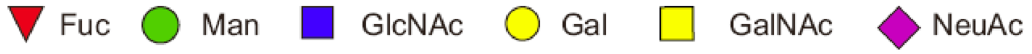

SUPPLEMENTARY FIGURE 3

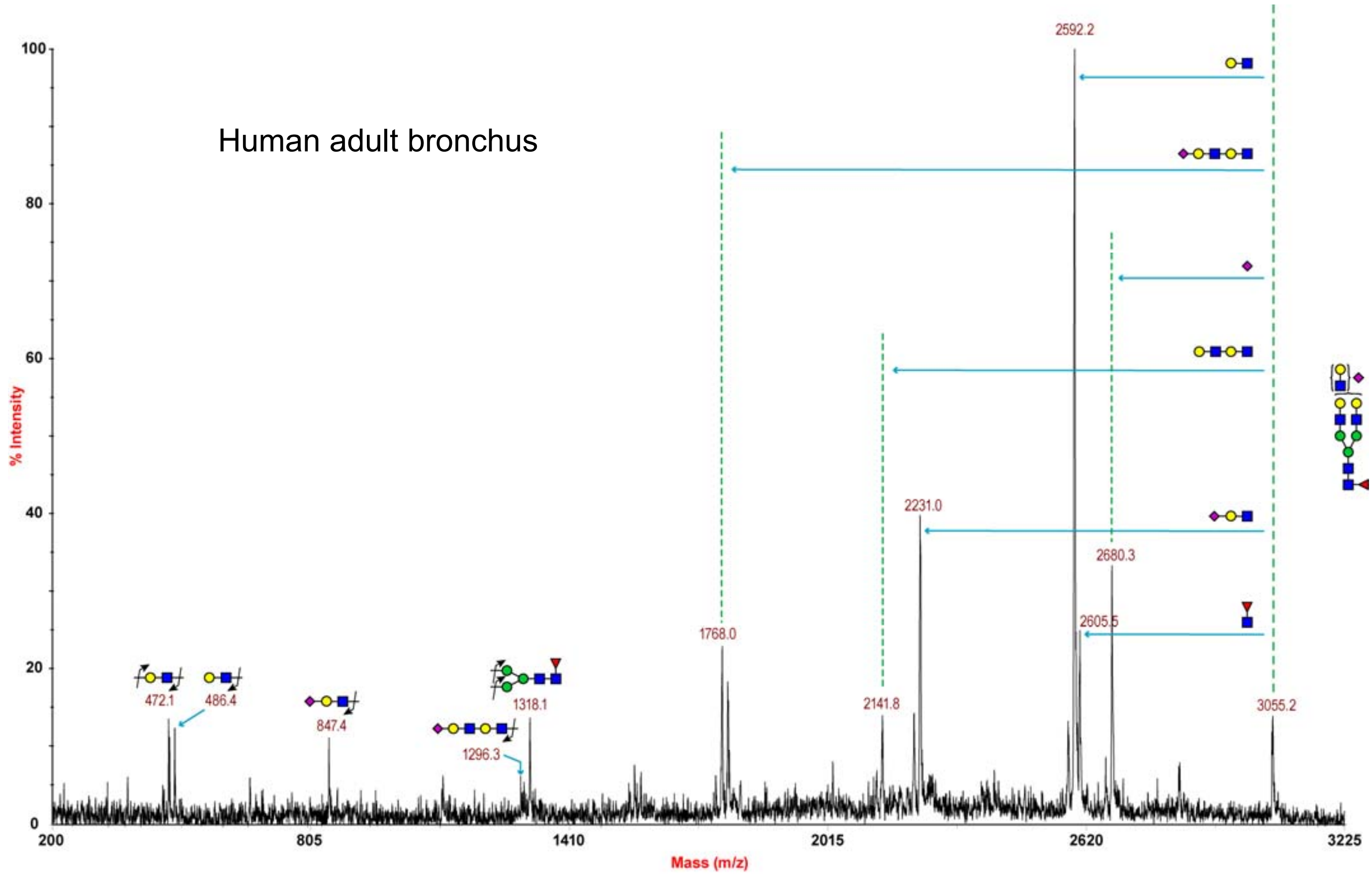

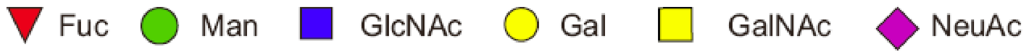

SUPPLEMENTARY FIGURE 4

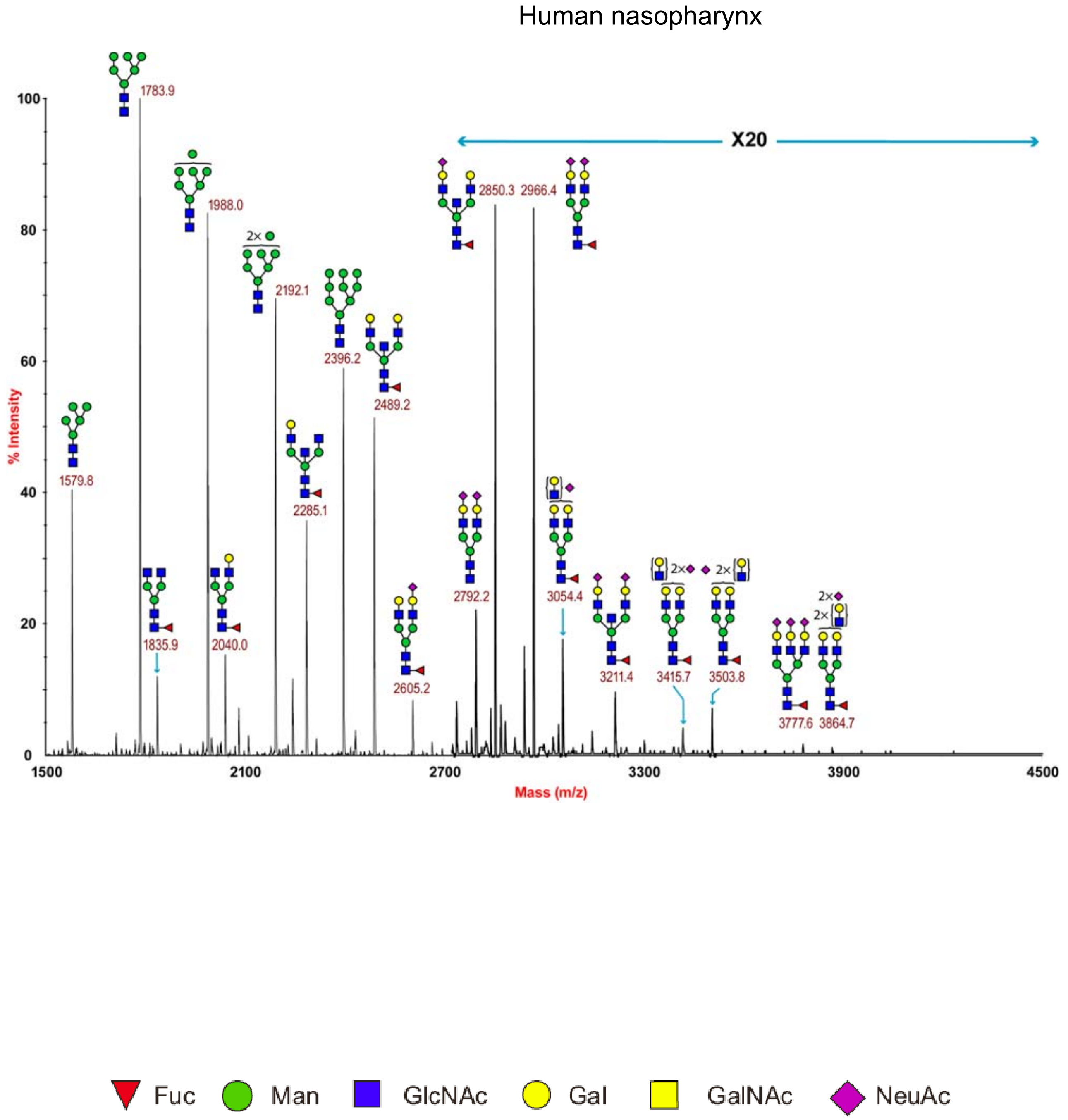

SUPPLEMENTARY FIGURE 5

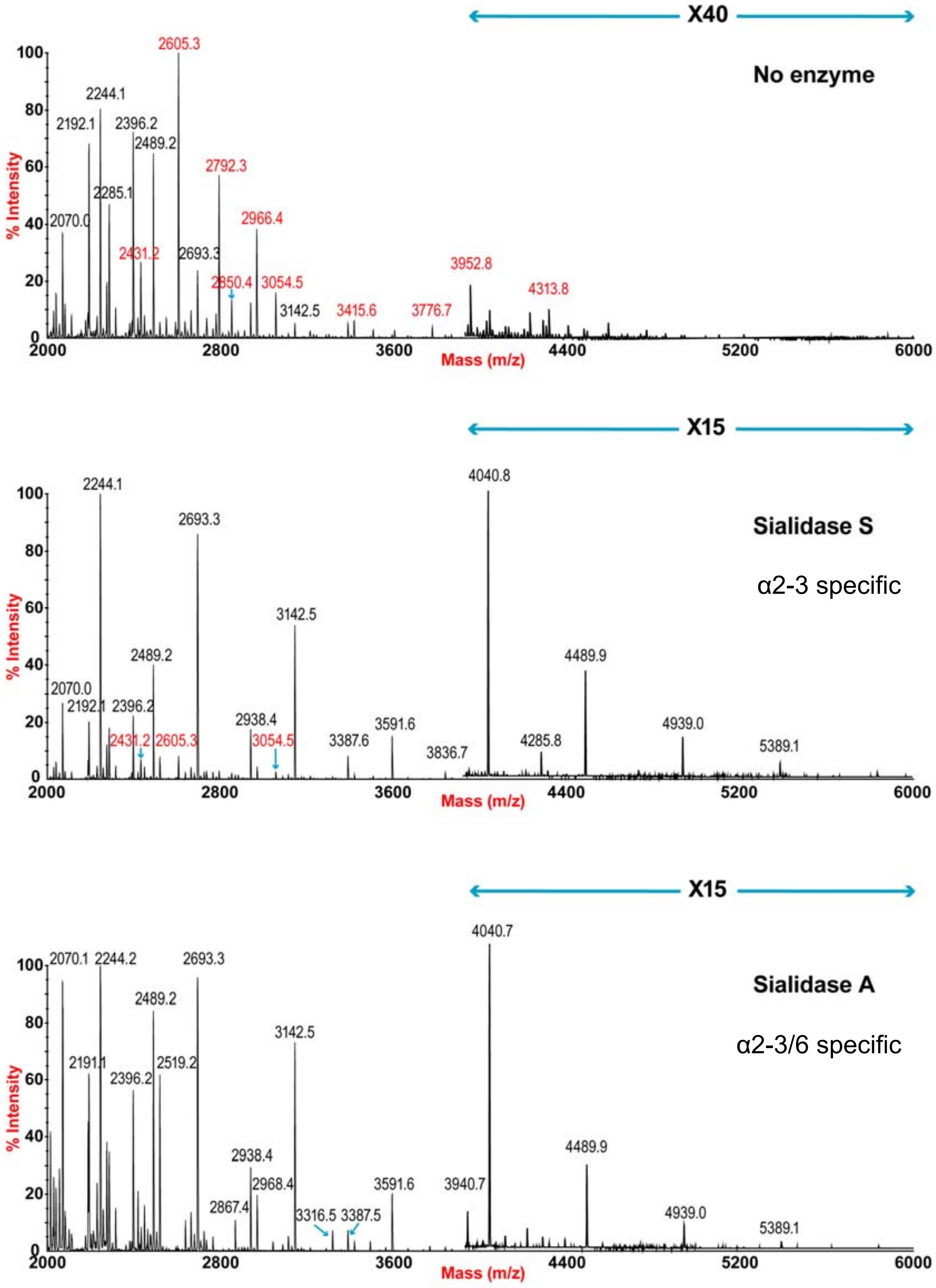

SUPPLEMENTARY FIGURE 6

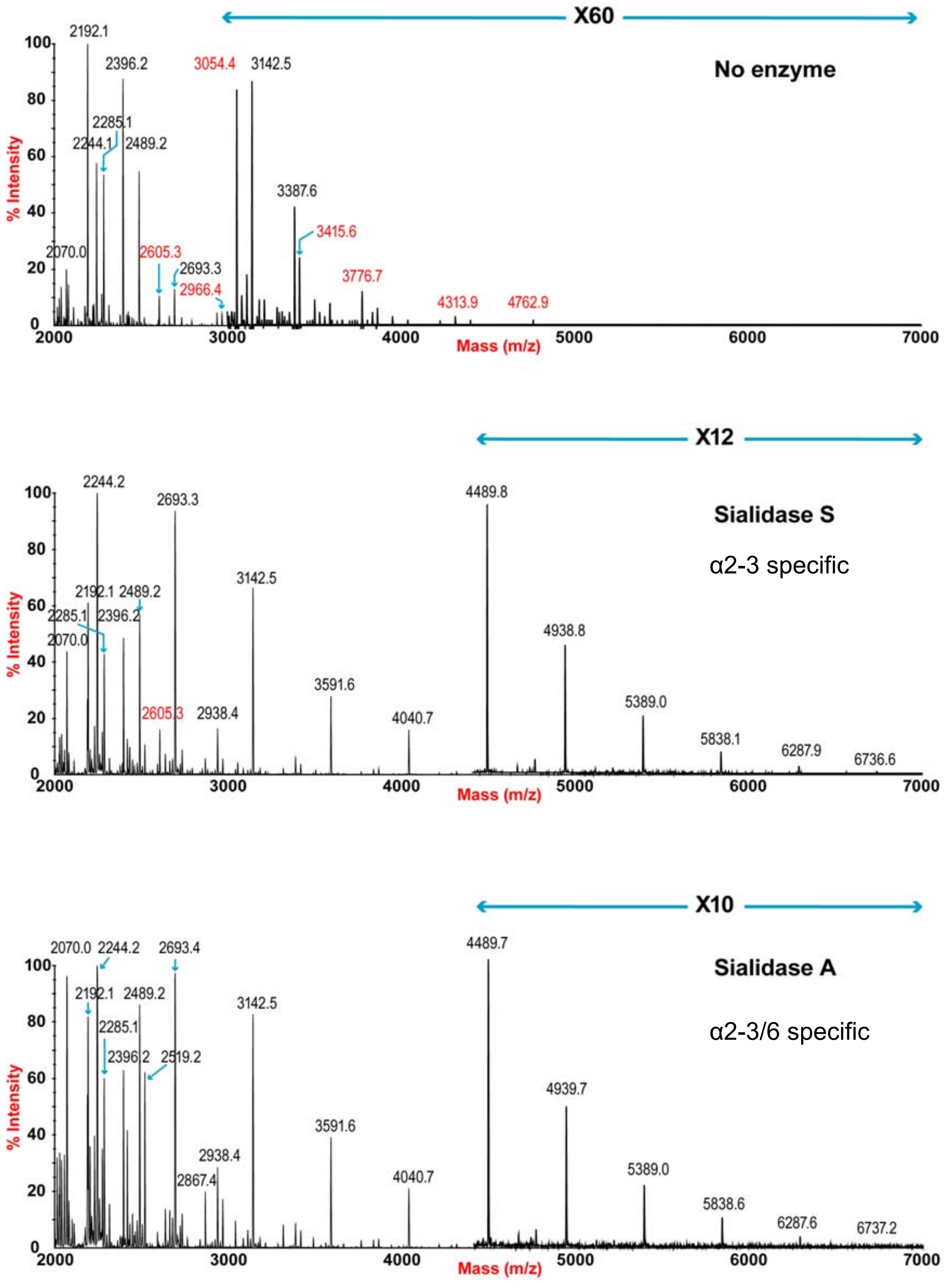

SUPPLEMENTARY FIGURE 7

LUNG

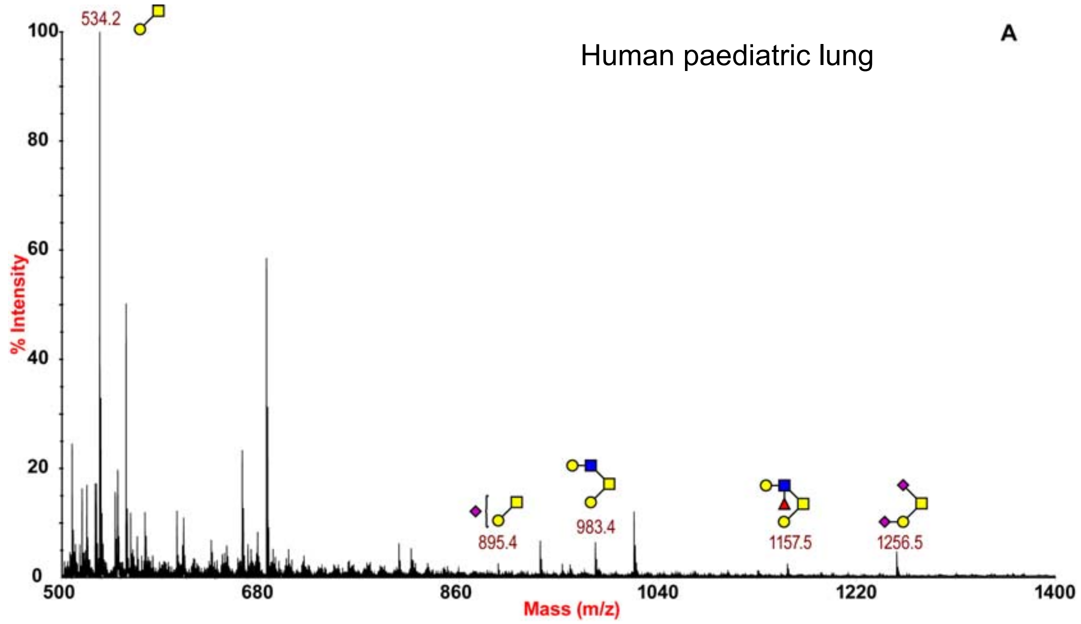

BRONCHUS

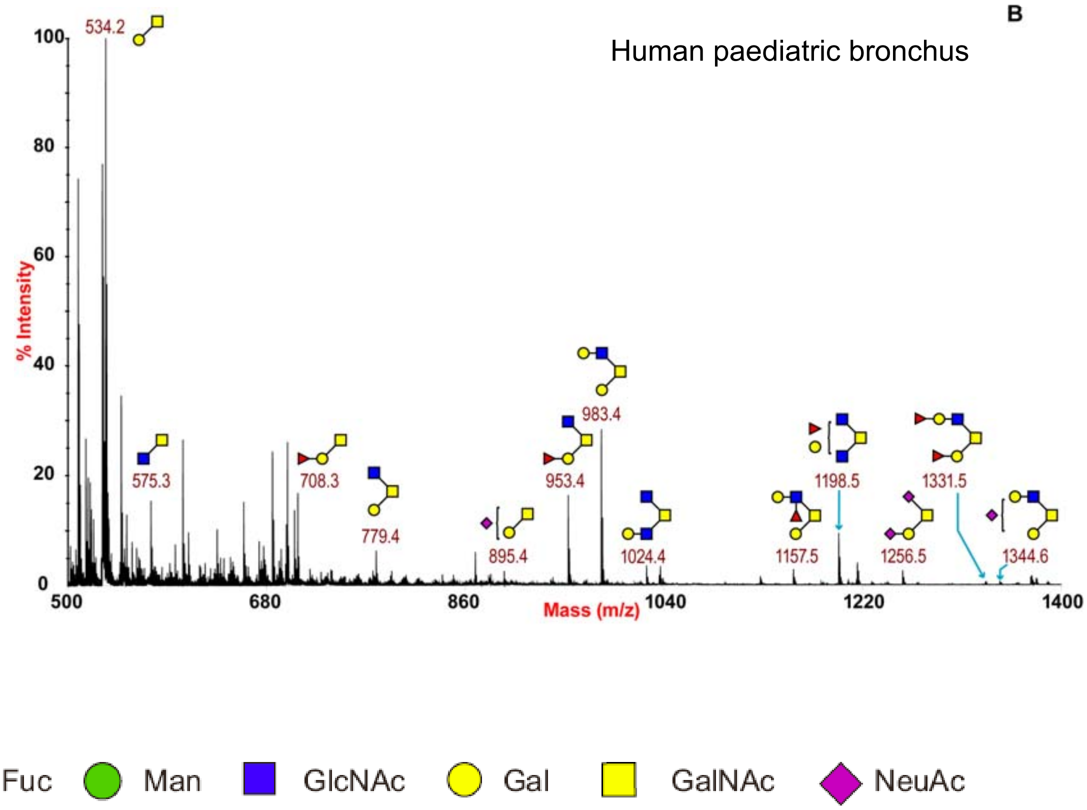

Supplement: Text S1 — Supplementary figures and tables. (PDF) [file ppat.1003223.s001.pdf]
